# Advances in intratumoral immunotherapy from a neuro-immune-endocrine perspective for breast cancer treatment

**DOI:** 10.3389/fonc.2026.1735300

**Published:** 2026-02-19

**Authors:** Claudia Angélica Garay-Canales, Mariana Segovia-Mendoza, Yair Rodríguez-Santiago, Karen Elizabeth Nava-Castro, María del Sol Ríos-Avila, Guadalupe Esther Ángeles López, Valeria Vargas Ponce de León, Diana L. Ruiz-Antonio, César Antonio Zavala-López, Carmen T. Gómez de León, Jorge Morales-Montor

**Affiliations:** 1 Neuroimmune Endocrine Interactions Laboratory, Instituto de Investigaciones Biomédicas, Departamento de Inmunología, Universidad Nacional Autónoma de México, Ciudad de México, Mexico; 2 Facultad de Medicina, Departamento de Farmacología, Universidad Nacional Autónoma de México, Ciudad de México, Mexico; 3 Laboratorio de Biología y Química Atmosférica, Instituto de Ciencias de la Atmósfera y Cambio Climático, Departamento de Ciencias Ambientales, Universidad Nacional Autónoma de México, Ciudad de México, Mexico; 4 Posgrado en Ciencias Biológicas, Unidad de Posgrado, Universidad Nacional Autónoma de México, Ciudad de México, Mexico

**Keywords:** breast cancer, hormone blockers, intratumoral immunotherapy, local delivery, neuro-immune- endocrine network (NIE) network, neurotransmitters, tumor microenvironment (TME)

## Abstract

The incidence rate of breast cancer continues to grow worldwide and is increasingly occurring in younger women. Although treatments have improved, they present several distinct challenges, as life-threatening side effects, relapse, metastasis, and ultimately death in women and men of productive ages. The immunotherapeutic strategies are focused on the postoperative context, and the systemic intravenous therapies have shown benefits in some patient populations with overall survivor rates higher in hormone-dependent breast cancer, but still high relapse rates, especially in more aggressive cancers such as HER2+ and triple negative. To halt tumor progression, it is necessary to identify all players involved, but most importantly, acknowledge the interactions of tumor cells with their surroundings. The tumor microenvironment (TME) has emerged as a conceptual framework that underscores the collective influence of cellular, structural, and signaling elements coexisting around the tumor. These components do not merely act as passive bystanders; rather, they form a dynamic milieu that shapes tumor behavior, therapeutic responsiveness, and disease trajectory. This reinforces the notion that effective interventions must address not only malignant cells but also the broader contextual landscape in which they evolve. While intratumor refers to heterogeneity within a single tumor, the TME encompasses the surrounding non-cancerous cells, molecules, and vasculature that interact with the tumor, collectively forming a dynamic landscape that modulates therapeutic responses and disease trajectory. The neuro-immune-endocrine (NIE) network plays a complex role in breast cancer, with the nervous system influencing tumor growth, immune evasion, and metastasis through neurotransmitters and neuropeptides. The endocrine system influences the TME through hormones such as estrogens, even in non-estrogen-dependent tumors in breast cancer. At the same time, immune cells interact with both neural and endocrine components, responding through cytokine release and phenotype modulation, thereby mounting a permissive or cytotoxic response to combat tumors. Stress, which activates the sympathetic nervous system, can affect immune cells and hormone release, impacting treatment adherence and positive prognosis. Nowadays, local intratumoral delivery with diverse mechanisms of action has been shown to mitigate systemic toxic effects and ensure targeted delivery, and has progressed to clinical trials, demonstrating promising outcomes. These mechanisms include, but are not limited to, triggering immune responses using pathogens, enhancing immune responses with recombinant cytokines, inhibiting immune checkpoints with monoclonal antibodies, or combining two or more of these strategies. Despite the high mortality associated with breast cancer in aggressive subtypes and its characterization by well-defined primary lesions, the clinical application of non-systemic intratumoral chemotherapy remains limited. In this review, we summarize effective intratumoral immunotherapeutic approaches for inhibiting tumor growth and/or metastasis in breast cancer. Specifically, we focus on the interactions within the NIE network that contribute to a sustained resolution of breast cancer. By elucidating these interactions, we aim to 1) predict treatment outcomes, 2) explain why some patients do not respond to innovative therapies, and 3) propose novel strategies for modifying the TME through targeted delivery of therapeutic agents with new materials. Furthermore, this approach paves the way for tumor-targeted modulation of other potential endocrine modulators, cytokine/chemokine delivery, and neurotransmitter modulation within the TME, representing a novel frontier in cancer treatment.

## 1 Introduction

**Figure 1:**
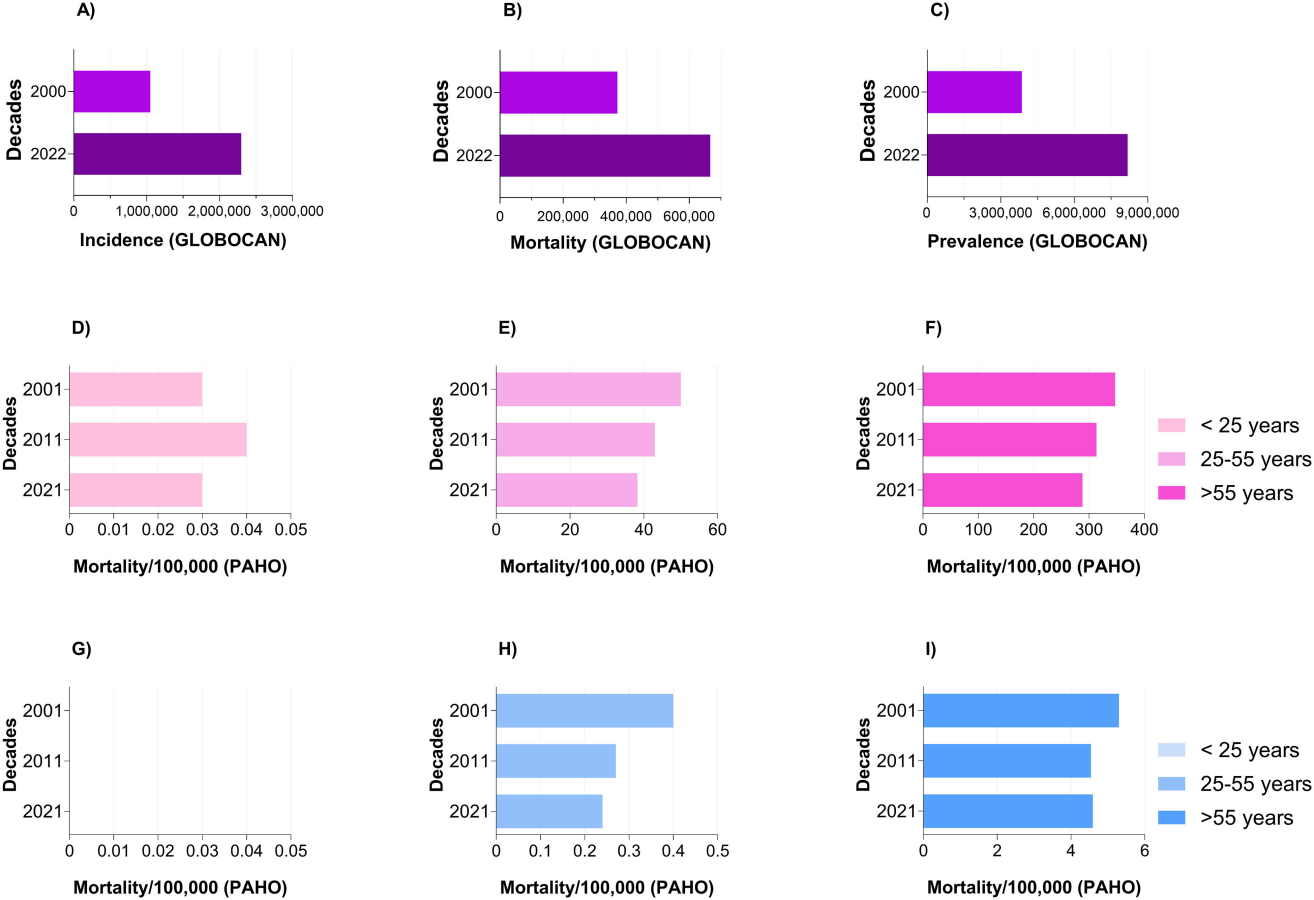
Global comparison of BC: **(A)** incidence, **(B)** mortality, and **(C)** 5-year prevalence among females, shown as case numbers in 2000 versus 2022. Data source: Global Cancer Observatory. **(D–I)** Breast cancer mortality in the Region of the Americas through three decades, 2001, 2011, and 2021, grouped by age and standardized by 100,000 individuals: females (pink bars) or males (blue bars) D/G) < 25 years, E/H) 25–55 years, F/I) > 55 years. Data source: Pan American Health Organization (PAHO).

In breast cancer (BC), uncontrolled cell proliferation leads to tumor formation and the development of a multifactorial disease. BC tumors may grow in different breast areas, such as lobules, ducts, and connective tissue, with ductal cancer the most common of all (1). According to the World Health Organization (WHO), BC is the most common cancer in prevalence (5-year: 8,178,393 cases), incidence [46.8 Age-Standardized Rate (ASR) per 100,000], and mortality (12.7 ASR per 100,000) (2). In 2022, approximately 2.3 million women globally received a diagnosis of BC, with 670,000 fatalities attributed to the disease. These numbers duplicated in incidence compared to 2000; the mortality did not duplicate in this period, but prevalence almost tripled in two decades (**Figures 1A–C**). BC can affect women in any nation and at any age following puberty, although its prevalence increases with advancing age.

Global statistics reveal substantial disparities in the impact of BC, contingent upon levels of human development. For instance, in countries with a very high Human Development Index (HDI), 1 in 12 women is likely to be diagnosed with BC during their lifetime, and 1 in 71 will succumb to it. In contrast, in nations with a low HDI, while only 1 in 27 women will receive a BC diagnosis in their lifetime, 1 in 48 will die. In 2020, America accounted for nearly 25% of newly diagnosed breast cancer cases. In Latin America and the Caribbean, a notably higher proportion of women, 32%, are diagnosed with the disease before the age of 50, in contrast to 19% in North America. Over 210,000 new BC cases were reported in Latin America and the Caribbean, accompanied by nearly 68,000 fatalities. In these regions, 50% of BC-related deaths occur in women under the age of 65, whereas in North America, this figure is 37% (3).

Additional risk factors include genetic predisposition, family history of BC, age, ethnicity, a higher body mass index (BMI), and having never given birth (4). Notably, pregnancy lowers the risk of BC by promoting the maturation of breast cells and reducing estrogen exposure during pregnancy (5). Factors that increase estrogen exposure include an early onset of menstruation, late menopause, and the use of 17β-estradiol (E2) and some derivatives in contraceptives or hormone replacement therapy (4). While predominantly affecting women, BC is also diagnosed in men. Male breast cancer (MBC) represents only a small fraction (approximately 1%) of all BC cases. Despite this low incidence, MBC cases have shown an increasing trend in recent years. Notably, male patients tend to have poorer outcomes than females. They are more frequently diagnosed at an advanced stage, experience lower survival rates, and have a worse overall prognosis (6, 7).

BC can be classified into four primary molecular subtypes using immunohistological techniques, based on the presence of the estrogen receptor (ER), the progesterone receptor (PR), and the human epidermal growth factor receptor 2 (HER2) (8): i) Luminal A BC, characterized by ER+ and/or PR+ and HER2-, makes up approximately 70% of BC cases and has a favorable prognosis (9); ii) Luminal B BC, defined by ER+ and/or PR+ and HER2+, accounts for 9% of BC cases, is associated with a high Ki67 level (> 14%), a marker of cell proliferation, and has a less favorable prognosis (7); iii) HER2 BC, identified by ER-, PR-, and HER2+, represents 4% of BC cases and is linked to a poor prognosis (8); and iv) triple-negative BC (TNBC), characterized by ER-, PR-, and HER2-, makes up 11% of BC cases and is known for its increased aggressiveness and poorer prognosis compared to other molecular subtypes, often impacting younger women (10, 11).

The 5-year relative survival rate for BC is encouraging, with a rate of 91.7% across all subtypes and stages. However, the 5-year relative cancer-specific survival rate for metastatic BC remains significantly lower at 32.6%, regardless of subtype, and can decrease to 11% for metastatic TNBC (Female Breast Cancer Subtypes—Cancer Stat Facts) (11). Additionally, the International Agency for Research on Cancer (IARC, WHO) projected that the number of BC-related deaths among women aged 10 to 85+ years from 2022 to 2045 is anticipated to rise significantly across various regions: In Asia, 121.8% in Oceania, 134.8%; Europe, 73.8%. In North America, 108.9%, Latin America and the Caribbean, 133.5%. Finally, in Africa, an increase of 189.8% (2). Previous data indicate that incidence is rising globally, with poorer overall survivor rates in low HDI countries. Meanwhile, mortality over three decades in men and women has been slowly decreasing in all age groups, despite the great efforts in early diagnosis and improved therapies (**Figures 1D–I**). This clearly indicates that current treatment strategies for patients with non-metastatic and especially metastatic BC are inadequate to achieve a satisfactory survival rate. Consequently, it is imperative to develop novel treatment approaches and take into account the connections of cancer cells with their surroundings for all BC, especially for metastatic TNBC. This review critically examines current intratumoral immunotherapeutic strategies for breast cancer, aiming to establish a conceptual and translational framework for designing targeted therapies that integrate biological, immunological, and systemic regulatory dimensions. Focusing on the neuro-immune-endocrine (NIE) perspective, we elucidate how the intricate interactions between tumor cells and the tumor microenvironment (TME) influence the efficacy of these strategies. Our goal is to identify mechanistic principles that can inform the development of next-generation localized interventions, which reprogram the TME based on immunoendocrine interactions, enhance antitumor immunity, and reduce relapse and metastatic dissemination.

## 2 Interactions of the NIE network and the TME in breast cancer

The NIE network, first described by Basedovsky in 1977, is a complex system of bidirectional communication among the nervous, immune, and endocrine systems, which play a critical role in the development, progression, and metastasis of breast cancer. These interactions influence the TME and the body’s homeostasis. In breast cancer, they modulate tumor initiation, growth, invasion, metastasis, reverse resistance to drugs, promote inflammation in tumors, and impair the immune system’s ability to combat cancer (12). Here, we examined the key components of the TME and the recent findings regarding the complex interaction with the NIE network that can both promote or inhibit tumor development, depending on the specific signaling pathway and the TME.

### 2.1 TME in breast cancer

Tumors are dynamic and heterogeneous systems that constantly evolve. Cancer cell progression depends on the interactions of tumor cells with numerous components within the microenvironment, facilitating suitable growth and resistance to immune cell control and treatment (13). Tumors are classified into “immune hot”, whose infiltrating immune population actively responds to various stimuli and enhances the therapeutic response to diverse agents, primarily those mediated by checkpoint blockade. On the other hand, there are also tumors whose immune infiltrate is anergic, which are called “cold tumors” and exhibit characteristics opposite to those of hot tumors (14). Immunotherapy can transform “immune cold” tumors into “immune hot” ones, improving treatment outcomes. Studying the TME in BC can help identify biomarkers to modulate the TME. For example, T-cell levels are associated with pathological complete response (pCR) and overall survival (OS) in BC patients. Traditionally, the components of the TME are studied separately, overlooking their interactions with NIE. Here, we provide insights that can inform predictions of neoadjuvant therapy efficacy and facilitate the development of novel BC classifications for survival prediction.

The TME is composed of tumor cells, immune cells (which are subject to polarization), stroma cells with transitional stages and reprogrammed features, cytokines and chemokines, hormones, proteins from the extracellular matrix, and other soluble factors. Among the immune cells within the tumor are macrophages, various subsets of lymphocytes, natural killer cells (NKs), neutrophils, myeloid-derived suppressor cells (MDSCs), dendritic cells, and innate lymphoid cells (ILCs). Initially, tumor cells create a hypoxic environment, which facilitates the epithelial-to-mesenchymal transition (EMT). However, an important recruitment of infiltrating immune cells occurs; yet, several mechanisms induce the suppression, exhaustion, or polarization of these immune cells, thereby promoting conditions that facilitate uncontrolled proliferation and tumor growth (15). The interactions of breast tumor cells with the TME are crucial to clinical outcome, influencing progression and resistance to treatment (16).

Once breast tumor cells are established, macrophages and dendritic cells activate T-cells through the stimulation of NF-κB, initiating the secretion of pro-inflammatory cytokines and upregulating nitric oxide synthase-2 (NOS2), which inhibits tumor cell proliferation. On the other hand, M2 macrophages and TAMs polarization begins upon the recruitment of circulating monocytes or resident macrophages when exposed to colony-stimulating factor 1 (CSF1), CCL2, IL-10, or TGF-ß, and the binding of the corresponding receptor, leading to hypoxic environment and production of Vascular Endothelial Growth Factor (VEGF) essential for tumor angiogenesis, Arginase 1 (ARG-1) responsible for L-arginine hydrolysis required for T cell and NK activation, Granulocyte-macrophage colony stimulating factor (GM-CSF) produced by tumor cells promotes programmed death ligand-1 (PD-L1) overexpression on TAMs inhibiting as well CD8+ (17–19).

**Figure 2:**
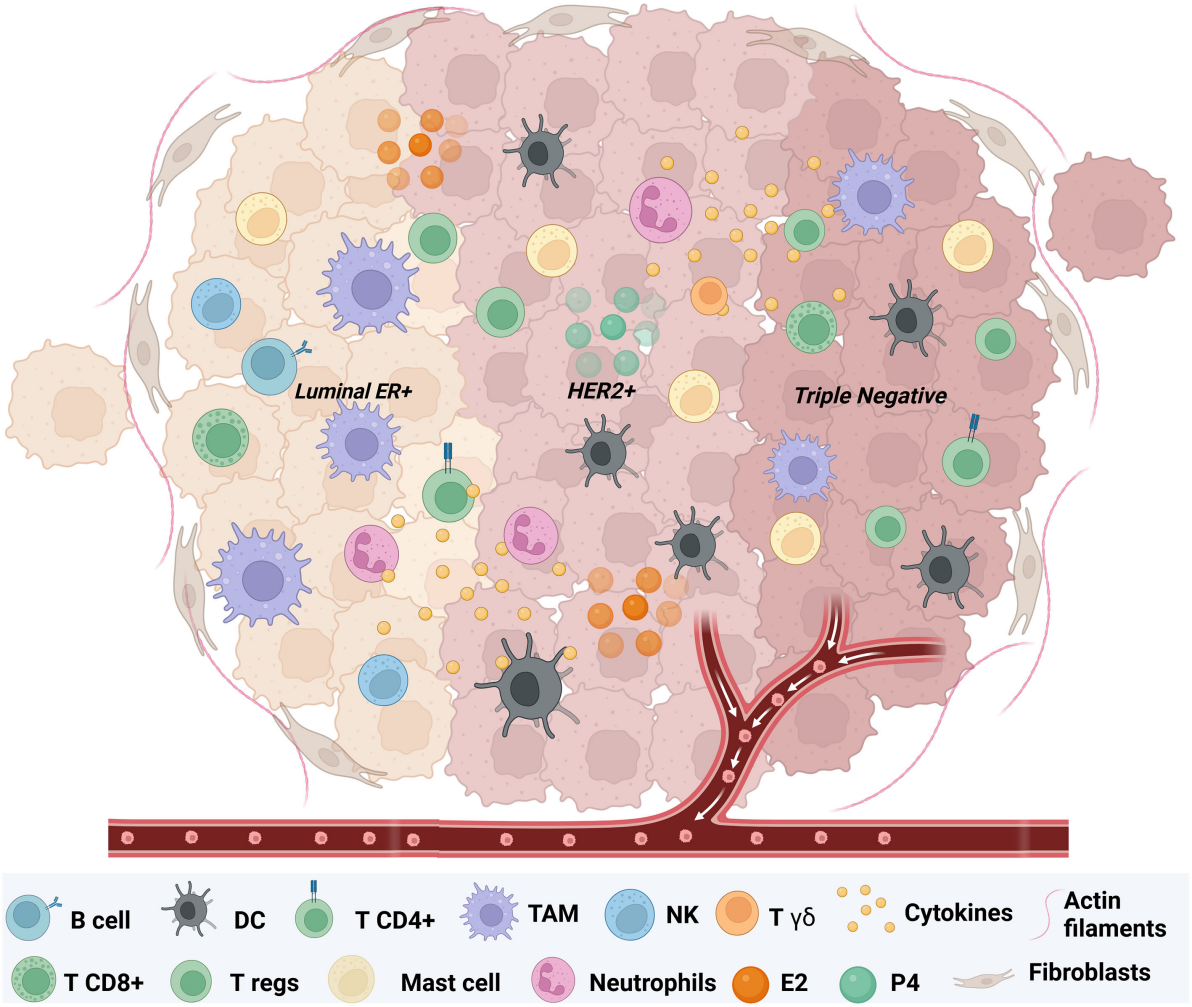
Representative images of the tumor immune microenvironment of different types of BC. Image created with Biorender.

The tissue-resident macrophages (TRMs) activate regulatory T cells (Tregs) when they encounter tumor cells, thereby initiating epithelial-mesenchymal transition (EMT). IL-10 abrogates cytotoxic activity of CD8+ lymphocytes by inhibiting IL-12 expression on dendritic cells, which in turn prevents the differentiation of T helper-1 (TH1) cells. The presence of numerous Tregs and few CD8+ cells is associated with a poor prognosis. Here, we highlight the features of representative cells from the TME of different breast cancer subtypes to better understand where intra-tumoral treatment options could be effective. **Figure 2**.

### 2.2 Cellular components of NIE involved in BC

#### 2.2.1 Macrophages and tumor-associated macrophages

Macrophages are a crucial component of the innate immune system, playing a vital role in defending against foreign pathogens and promoting specific immunity. However, they may play a dual role within the TME of BC: macrophages may play as “friends” during early stages of the carcinogenic process and as “foes”, later in the process, when they became tumor-associated macrophages and facilitate a pro-tumoral response -associated with enhanced tumor progression and the promotion of cancer cell growth, angiogenesis, and immunosuppression-. This paradoxical role of macrophages in breast cancer is attributed to their plasticity and ability to adopt different phenotypes, namely M1, M2, M3, M4, Mreg, oxidative macrophages (Mox), and M17, that instead of being a determined cell lineage, constitute a spectrum of cells with a particular secretion and function profile responding to the environmental conditions (20); that may or may not, contribute to breast cancer progression.

M1 macrophages are characterized by their anti-tumor properties, which promote T cell recruitment and activation, and induce the production of pro-inflammatory cytokines such as IL-12 and TNF-α (16). In contrast, M2 macrophages exhibit pro-tumor properties, promoting immunosuppression, angiogenesis, and tumor cell growth. The balance between M1 and M2 macrophages within the TME is critical in determining the outcome of breast cancer. Studies have shown that a high density of M2 macrophages within the TME is associated with poor prognosis, metastasis, high histological grades, low neoadjuvant chemotherapy response, and reduced survival rates, especially in triple-negative breast cancer patients (21).

Moreover, the expression of estrogen receptors (ER) in macrophages and monocytes has been shown to modulate their actions and metabolism, with E2 treatment promoting the adoption of the M2 phenotype, meanwhile induces regulatory T cells (Treg), upon the secretion of IL-10, transforming growth factor (TGF-ß), and CCL18 (16). As mentioned above, macrophages and even TAMs are cells with high plasticity capable of differentiating into several subsets of regulatory, active, or suppressive cells. A deeper understanding of these subsets, particularly in terms of function and secreted molecules, enables the strategy to target these cells, thereby modifying their recruitment within the tumor and activating or repolarizing them to an anti-tumoral phenotype.

#### 2.2.2 Lymphocytes

Tumor-infiltrating lymphocytes (TILs) play a crucial role in the tumor immune microenvironment. At the onset of BC, the presence of TILs often indicates a strong immune response, and the quantity is associated with a favorable prognosis (22). Cytotoxic (CD8+) T cells are recognized as the primary immune cells responsible for identifying and killing tumors (23). According to the meta-analysis of Sun et al, a high CD8+ T-cell infiltration level was significantly related to better overall survival and relapse-free survival. Furthermore, these high levels of infiltration were associated with decreased expression of estrogen and progesterone receptors, as well as increased expression of human epidermal growth factor receptor 2 (HER2) in these patients (24).

At early stages, CD4+ T helper (Th) cells coordinate the adaptive response at epithelial sites by releasing a wide array of cytokines and recruiting other immune cells. The Th cell subsets include Th1, responsible for the cell-mediated immunity through the production of IFNγ, TNF-α, and other inflammatory mediators, directly destroy cancer cells and facilitate the recruitment and activation of CTLs (Cytotoxic T lymphocytes), and NK cells (25). In contrast, Th2 cells are associated with tumor promotion through chronic inflammation by activating the humoral immune response and decreasing the recruitment and activation of CTLs (26). However, epidemiological studies found that allergic patients with a strong Th2 response are less susceptible to developing BC (27). The observation indicates the induction of terminal differentiation in cancer cells through thymic stromal lymphopoietin, causing the epigenetic reprogramming of the tumor cells, activating mammary gland differentiation, and suppressing EMT, thereby blocking breast carcinogenesis by secreting IL-3, IL-5, and GM-CSF (28).

#### 2.2.3 NKs

The role of NKs in BC involves the tumor cell recognition and elimination through soluble factors, including cytotoxic granules, proinflammatory cytokines, and chemokines (29). In advanced stages of BC, NKs are significantly modulated in different parameters, including absolute number, cytotoxicity, and lytic capacity toward K562 cells. High activity of peripheral NK (pNK) is associated with an effective response to neoadjuvant chemotherapy, overall survival, and disease-free survival (30). NKs interact with multiple immune cells: a) CD8+ can enhance the function of NKs through IFNs, TNF-α, and IL-12, and reciprocally, NKp44+ NK cells can also affect the clonality of CD8+ T cells; b) DCs augment NK function by secreting IFN- and TNF-α and regulating the TLR7/mROS/IL12 axis; c) T regs CXCR4+ inhibit NK activation, releasing GZMB and CD107a; d) MDSCs impair NK cell cytotoxicity by overproducing inducible nitric oxide synthase (iNOS), which generates nitric oxide (NO) from L-arginine. This NO affects mechanisms such as FcR function and IFN- γ (31, 32); e) TAMs inhibit activation of CD69 and TGF-ß maturation (33). Thus, NKs secrete cytokines to promote cell apoptosis, inhibition of tumor cell proliferation, and angiogenesis (34).

#### 2.2.4 Dendritic cells

DCs interact with T lymphocytes and regulate their activation by presenting antigens and producing specific cytokines. DCs are classified into a) myeloid, involved in immune cell activation, and b) plasma-like cell populations that can produce IFN-I, which is associated with poor prognosis (35). At the early stages of BC, DCs migrate to lymphoid organs, where CD40 on DCs and CD40L on T cells deliver antigens to immature T cells in lymphoid organs, activating CD4+ helper T cells and CD8+ T cells. This process induces the production of IFNs and the stimulator of interferon genes complex (STING). The production of interferon and the recruitment of DCs promote antitumor immunity. A key activity of DCs is the uptake of tumor cell DNA and the activation of T cells. A high percentage of DCs in the blood constitutes a favorable prognosis factor; however, upon cancer progression, tumor cells can inhibit the maturation of tumor-infiltrating DCs, resulting in a low presentation of antigen and downregulated expression of co-stimulatory molecules. Currently, DCs loaded with tumor antigens can more effectively target the tumor. The training of these DCs can be performed *in vitro* or by engineering DCs to express tumor-associated antigens and later introducing them into the patient. These modified DCs, along with proper cytokines, can enhance the T-cell mediated destruction of tumors (36).

#### 2.2.5 Marrow-derived suppressor cells

MDSCs are immature bone marrow cells that can promote the proliferation of regulatory T cells and inhibit the activity of cytotoxic T lymphocytes. These cells drive tumor growth by reprogramming BC. The higher numbers of MDSCs infiltrating the tumor are a higher risk factor for tumor progression. BC can promote differentiation and recruitment through the secretion of granulocyte colony-stimulating factor (G-CSF), macrophage colony-stimulating factor (GM-CSF), and the chemokine CCL3. Also, the MDSCs secrete IL-10, TNF-α, and VEGF, supporting the activation of EMT and angiogenesis (37). As mentioned above the NKs are impaired of NO production by MDSCs allowing tumor progression. On the other hand, the influence of neuropeptides in the response to treatment are revealed by studies investigating the effects of Substance P (SP), a sensory neuropeptide, on breast cancer metastasis and radiotherapy (RT) response showed that depleting neuropeptides or removing vagus nerve input increased metastasis of triple-negative breast carcinoma. Continuous exposure to low-dose SP reduced tumor-infiltrating myeloid-derived suppressor cells and TNF-α response to LPS challenge, increased CD4+CD25+ cells and IFN-γ secretion in lymph nodes and spleen, and prevented tumor-induced sensory nerve degeneration and altered angiogenic factor release from cancer-associated fibroblasts and tumor explants (38).

#### 2.2.6 Cancer-associated fibroblasts

CAFs play a crucial role in modulating the TME, supporting the survival, angiogenesis, immunosuppression, and treatment resistance of cancer cells. CAFs release fibronectin, TGF-β, and laminin, allowing a looser environment for metastatic BC cells, especially in TNBC patients, where high activation leads to lymph node metastasis, infiltration, and polarization of M2 macrophages (39). These cells are highly expressed in the tumor and mediate estrogen-independent tumor growth by selectively regulating ER-α signaling, suppressing estrogen responsive genes leading to therapeutic resistance, basal-like differentiation, and invasion. The targets are TFG-β and Janus kinase signaling cascade. Genes down regulated by CAFs in cancer cells were predicted with poor response to endocrine treatment (40).

#### 2.2.7 Mesenchymal stem cells

MSCs support cancer progression by promoting BC proliferation, EMT, and facilitating the de-differentiation of cancer cells into tumor stem cells, which leads to drug resistance, invasion, and immune escape. MSCs secrete IL-6, triggering the release of prostaglandin E2 (PGE2) and recruiting additional MSCs, meanwhile facilitate the invasiveness of BC (41). As many cells with dual role in tumor progression, MSCs can sensitize cancer cells to chemotherapy and radiotherapy, since these cells possess inherent regenerative and homing properties becoming candidates for cell-based therapies, since MSCs can be engineered to express therapeutic molecules or deliver anti-cancer agents directly to tumor sites.

#### 2.2.8 Tumor-associated neutrophils

At the onset, neutrophils infiltrate breast tumors, becoming highly activated, which promotes inflammation and antitumoral activity (known as the N1 subtype). IFN-α/γ promote the production of N1 type. Later, TGF-β induces the production of N2-type with tumor progression phenotype and is related to poor prognosis. In mice, TANs N2 type recruit immunosuppressive cells and reduce the proliferation of CD8+ cells (42).

### 2.3 Soluble factors modulating the NIE network

All the components of the tumoral microenvironment communicate through soluble factors, including cytokines, chemokines, growth factors, hormones, and even neurotransmitters and these interactions reshape the response to therapeutic agents (43–47).

#### 2.3.1 Cytokines

The immune cells orchestrate tumor progression by releasing cytokines and chemokines, which may have either anti- or pro-tumor effects. Indeed, the presence of specific cytokines has been proposed as a marker of a better or worse prognosis and survival. The pro-inflammatory cytokines associated with an antitumoral environment are IL-2 and IL-12. In contrast, the overexpression of the proinflammatory cytokines such as TNF-α, IL-1β, IL-6, IL-17, IL-23, as well as the anti-inflammatory cytokine IL-10, is associated with a worse prognosis (43). Interestingly, the effect of these inflammation mediators can be harnessed as a treatment for BC, as is the case with TNF-α (48). The endogenous TNF-α is a potent pro-inflammatory cytokine. It promotes survival and proliferation of cancer cells (49) as well as tumor aggressiveness (50) and migration (51). Nonetheless, the exogenous administration of TNF-α induces the destruction of tumor vasculature and tumor cell necrosis (52), positioning itself as a potential therapeutic agent, but its high toxicity prevents systemic administration.

The role of multiple cytokines and chemokines in the BC TME has been elucidated and extensively reviewed elsewhere (43, 47). IL-6 is present in the TME from the initiation of the tumor, as well as IL-1β. The latter is released by multiple cells, including activated M2-type macrophages and TAMs. Meanwhile, IL-1β increases immune cell recruitment of macrophages and adaptive LTs CD4+ and CD8+, and promotes the release of IL-6 and IL-17 (53, 54). Additionally, IL-6 and IL-1β B, as well as TNF-α and IL-17, increase and maintain the aggressive phenotype of the tumor, participating in the EMT process, metalloproteinase expression, cancer stem cell renewal, cell migration, tumor growth, and angiogenesis.

The immune system has antitumoral responses mediated by cytokines. IL-12, IL-2, and IFNγ potentiate those responses. The high expression of IL-12/STAT4 is associated with a better prognosis and prolonged survival (55), presumably due to its ability to modulate the NKs, LT CD8+, macrophages, and Th1 CD4+ cells (56, 57). Additionally, it induces autophagy in tumor cells through the PI3K/Akt pathway (58). IL-2 and IFN-γ are also capable of modulating the differentiation of CD4+ and CD8+ cells into Th1 and Th2 cells (16, 59, 60) and enhancing their cytotoxic activity against cancer cells (61, 62). However, the levels of the cytokine are determinant for its effect on the tumor. Meanwhile, high levels of IFN-γ are anti-tumoral, and low doses can enhance the tumor progression and resistance to immunotherapy (43, 63, 64).

#### 2.3.2 Chemokines

Chemokines regulate cell recruitment and interleukin release in normal and pathological conditions (65). In the different subtypes of breast cancer, many chemokines are deregulated following different patterns (66). CXCL12, for example, is downregulated in basal tumors; meanwhile, it is overexpressed in luminal A tumors. CXCL12 is released by breast cancer cells and, together with its receptors CXCR4 and CCR7, promotes the tumor invasiveness, angiogenesis, and metastasis (67–69). CCL2, CCL25, and CCL10 are also involved in metastasis (70–72). Furthermore, as some chemokines are overexpressed in the plasma of breast cancer patients, they could act as predictors of breast cancer. CXCL8, CXCL9, and CL22 have been proposed as plasma biomarkers. Particularly, CXCL8 and CCL18 are indicators of advanced-stage carcinomas (73). In the case of chemokines, CXCL9 plays a dual role in BC, being antitumoral in TNBC but pro-tumoral in the luminal A subtype (47).

Chemokines and interleukins also communicate bidirectionally with hormones, active molecules in hormone-dependent tumors. In this regard, E2 downregulates CXCR7 and upregulates the expression of CXCR4. Hence, E2-dependent breast cancer growth would be promoted by E2 via CXCR4 and CCL12 (74). Similarly, in ER+ cancer, E2 increases the extracellular levels of CCL2 and CCL5, leading to macrophage infiltration. Moreover, when those chemokines are inhibited, the cancer cell growth and macrophage infiltration are diminished (75). Additionally, estrogens such as E2 regulate the differentiation, proliferation, and function of dendritic cells, macrophages, mast cells, and neutrophils, thereby shaping the composition of the TME (16). The effect of the hormone depends not only on the dose, but also on time intervals, as is the case with NK cells. E2 reduces the cytotoxic activity of NK cells in a dose-dependent manner (76). Notably, E2 administration has been shown to suppress NK cell activity at short time intervals, whereas longer intervals of exposure enhance NK cell activity (77).

#### 2.3.3 Steroid hormones

The development and physiology of the mammary gland are highly hormone-dependent (78). In BC, there are different patterns of ERα and ERβ, HER2, and PR expression, which mediate several processes during cancer progression (79). Indeed, higher levels of serum estradiol and testosterone are risk factors for BC (80, 81). Moreover, the expression of the aromatase, the enzyme that converts androgens into estrogen, is higher in BC (82). The activity of the aromatase is promoted by various components of the TME, including p53, prostaglandin E2, cyclooxygenase-2, TNF-α, IL-11, and IL-6 (83). It has been observed that estrogens and their quinone metabolites possess genotoxic, mutagenic, transforming, and carcinogenic potential, leading to abnormal cell proliferation (81, 84, 85). The precise role of estrogen and other hormones in the TME is complex and influenced by various factors, including cancer subtype, patient age, cellular receptors, dose, time, and cancer stage, among others.

Hormones, particularly E2 and progesterone, influence the TME through multiple interactions with stromal cells, immune cells, and extracellular matrix (ECM) (83). The E2 role as an immunomodulator has been reviewed previously (16, 83). E2 and P4 modify the ECM composition (86, 87). In this regard, E2 promotes angiogenesis (88), cell proliferation, migration, and growth, increases the number of cancer stem cells (89, 90), as well as tumor-initiating cell renewal (91, 92), and chemotherapy resistance (93–95). Together, cytokines, chemokines, hormones, and their receptors collectively orchestrate the tumor-supportive microenvironment, making them critical targets for therapeutic intervention (79, 96).

#### 2.3.4 Modulatory effect of neurotransmitters

The Central Nervous System (CNS) plays an immunomodulatory role through peripheral nerve endings, using soluble molecules known as neurotransmitters, which are broadly classified into two categories: small-molecule neurotransmitters and neuropeptides, both of which impact BC outcome. The immune system, both innate and adaptive, fights against cancer cells, but it is important to acknowledge that the immune cells involved are producer/sensitive to neurotransmitters, for instance, macrophages, T cells, and neutrophils can synthesize catecholamines in an autocrine/paracrine manner after an acute sympathetic stress reaction; however, chronic stress can lead to suppression of the activity of immune cells. On the other hand, dopamine, a neurotransmitter that inhibits stress, can also modulate immune responses, inhibit tumor angiogenesis and endothelial progenitor cell mobilization, and activate tumor immunity by suppressing MDSC activation and M2 macrophage polarization. Norepinephrine (NE) and epinephrine (E) stimulate angiogenesis, inhibit immune function, including NK cell cytotoxicity, dendritic cell maturation, and lymphocyte response, which in turn allows progression of the tumor (97).

Acetylcholine promotes cancer cell proliferation, migration, invasion, anti-apoptosis, stemness, and EMT; also increases macrophage recruitment, anti-inflammatory response and endothelial angiogenesis. Like acetylcholine, serotonin enhances the proliferation, migration, invasion, and autophagy of cancer cells, as well as the polarization of M2 macrophages, platelet activation, and angiogenesis. In contrast, Gamma-aminobutyric acid (GABA) can either increase (via GABAaR) or inhibit (via GABAbR) the proliferation, migration, or invasion of cancer cells, depending on the receptor expressed on the cell (44).

Beta-adrenergic receptors (β-ARs) are expressed in various immune cells, including T lymphocytes, B lymphocytes, and NK cells. Activation of β-adrenergic receptors (β-ARs) generally inhibits the response of these immune cells. CD8+ T cells, which are crucial for adaptive immunity, express more β2-ARs than CD4+ T cells. The activation of β2-ARs suppresses the function of CD8+ T cells, including their proliferation and cytolytic killing capacity. In addition to suppressing lymphocyte function, norepinephrine can also downregulate anti-tumor responses by promoting the accumulation of immunosuppressive cells. Beta-adrenergic signaling can also decrease the glucose uptake of T cells, contributing to stress-induced immunosuppression. The regulation of NK cell function is closely related to the sympathetic nervous system (SNS) and can be influenced by factors such as circadian rhythms, exercise, and stress. Infiltration of NK cells into TME depends on epinephrine and the secretion of IL-6; additionally, they can inhibit the function of cytotoxic T lymphocyte and NK cells along with prostaglandins. The mobilization of CD11b^+^ macrophages into the TME, which secrete NE, could increase metastasis in the lungs and lymph nodes.

Breast cancer cells often overexpress prolactin, which contributes to tumor growth, metastasis, and chemoresistance. Since dopamine inhibits prolactin production, researchers have investigated whether dopaminergic drugs can improve breast cancer outcomes (98, 99). Dopamine, a catecholamine neurotransmitter, regulates behavior, movement control, endocrine, and cardiovascular function. Studies suggest that dopamine or its receptor agonists may inhibit tumor growth in breast and colon cancers. However, the effectiveness of dopamine varies depending on tumor type, receptor expression, and dosage, failing to decrease the proliferation *in vitro* of breast cancer cell lines; Dopamine receptors are expressed on various immune cells, and their activation can either stimulate or inhibit the immune response (98). One key mechanism underlying dopamine’s tumor-suppressive effects is decreasing angiogenesis. Activation of the DRD1/cGMP/PKG pathway can induce growth arrest and tumor shrinkage, as well as reduce bone metastasis in breast cancer (100).

Serotonin (5-Hydroxytryptamine (5-HT)), synthesized by neurons and enterochromaffin cells in the intestine, plays a role in immune signaling and breast cancer progression. Serotonin can modulate macrophage polarization through 5-HT_2B_R and 5-HT_7_R (97). Research has shown that serotonin receptors, particularly 5HTR2A and 5HTR3A, are overexpressed in breast cancer tissues compared to normal tissues. Serotonin promotes angiogenesis by enhancing the expression of TPH1 (tryptophan hydroxylase 1) and VEGF, which supports tumor growth and metastasis. Studies have also demonstrated that serotonin stimulates the growth and division of breast cancer cells, including MCF-7 and MDA-MB-231 cells, through specific serotonin receptors (5-HT2A and 5-HT7) (101).

Neurotensin receptor-1 is overexpressed in approximately one-third of primary breast tumors (102). Activation of this receptor promotes tumor cell proliferation, invasion, and migration, while inhibiting apoptosis. Estrogen upregulates neurotensin in normal breast cells. In breast cancer cells, overexpression of the NTS-1 receptor is linked to increased migration and invasion. High NTS1 receptor expression is associated with poorer prognosis, including higher tumor grade, larger size, and increased lymph node metastasis (103). Additionally, neuropeptide Y and its receptors have been implicated in breast cancer progression and metastasis. Neuropeptide Y promotes angiogenesis, tumor cell proliferation, and metastasis via its effects on vascular smooth muscle and VEGF. Its receptors are overexpressed in various tumors, including breast cancer metastasis. Targeting the Y1 receptor may be a promising strategy for treating breast cancer and osteoporosis (104).

These findings suggest that neurotransmitters play a crucial role in breast cancer development and progression. The interactions should be studied to overcome treatment resistance.

## 3 Therapeutic strategies for BC

### 3.1 Classic treatments

**Figure 3:**
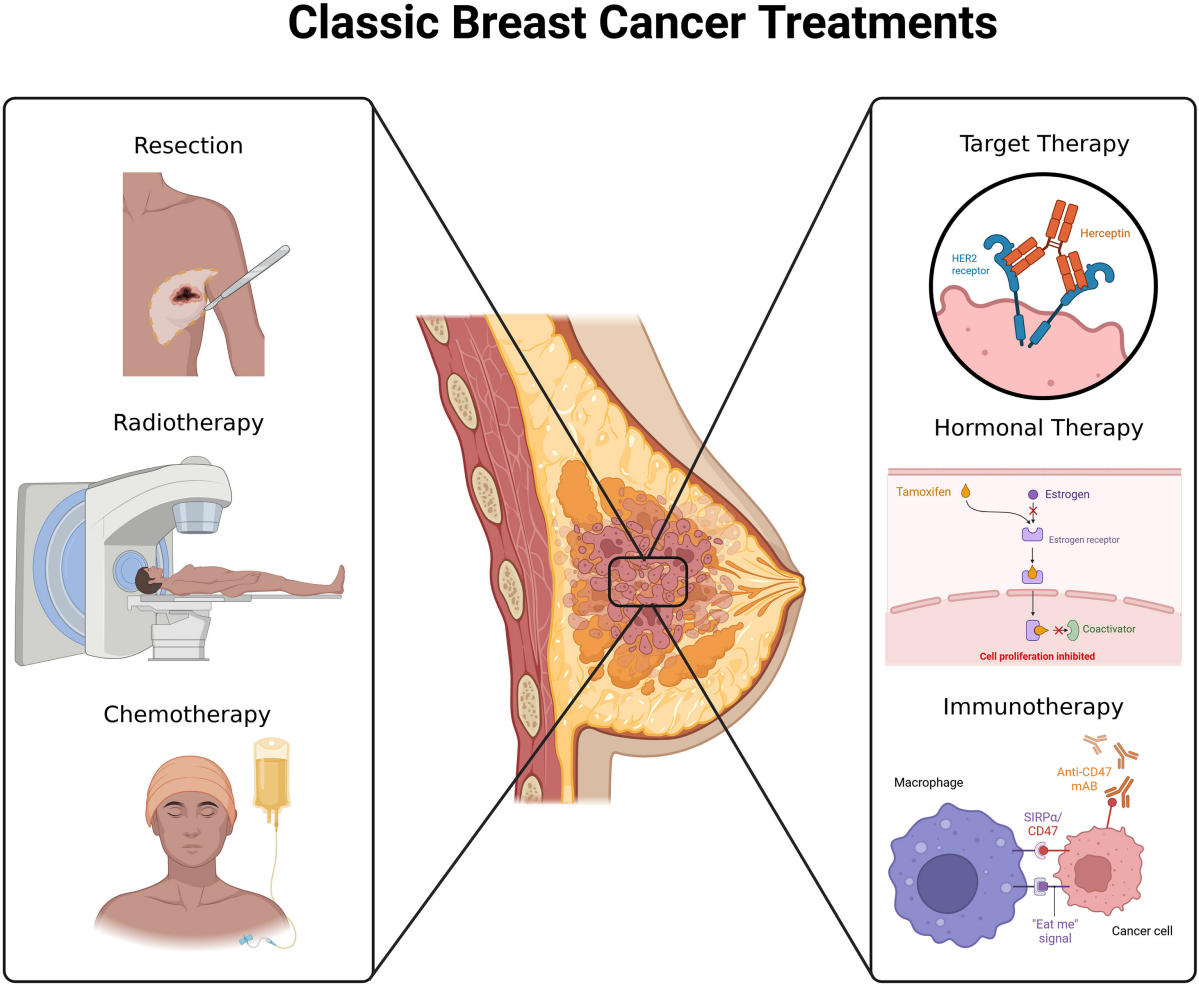
Classic therapeutic strategies used in BC. Image created with Biorender.

Classic breast cancer treatments refer to therapeutic approaches that have been used for decades due to their proven effectiveness and to serve as the first line of defense. Many of these treatments are systemic, allowing them to target the entire body to eradicate all tumor cells. Their effectiveness depends on the stage at diagnosis, treatment, the cancer subtype, and the patient’s intrinsic characteristics (**Figure 3**).

#### 3.1.1 Resection

Tumor resection, a surgical procedure involving the removal of tumors and surrounding tissue, has a history dating back to 1600 BC. The modern era of breast cancer surgery began in the 19th century with William Halsted’s radical mastectomy technique, which significantly reduced local recurrence rates. However, this approach was highly invasive, resulting in substantial physical and psychological sequelae (16, 105). Subsequent studies demonstrated that less invasive procedures, such as lumpectomies accompanied by adjuvant therapies, were as effective as radical mastectomies. Modern breast-conserving surgery (BCS) techniques prioritize tumor removal with clear margins, reducing recurrence risk while preserving cosmetic appearance and alleviating psychological burden (106). Resection is not considered a systemic treatment, but significantly impacts overall disease management, provides essential pathological data guiding subsequent treatment decisions, and, when combined with other therapies, reduces cancer risk, improving patient outcomes and quality of life (107).

Survival rates for breast cancer vary significantly depending on age, stage, and tumor type. Retrospective studies have consistently shown that patients with stage I-III breast cancer who undergo breast-conserving therapy (BCT) with radiotherapy and/or chemotherapy exhibit higher survival rates compared to those treated with mastectomy alone, regardless of age or hormone receptor status (108–110). Notably, postmenopausal women with stage I tumors benefit substantially from BCT accompanied by other therapies; however, older patients are more likely to be treated with mastectomy or BCT alone, a trend observed across multiple studies (111–113). Regardless of the surgical approach, resection remains the initial therapeutic step, although it may not be sufficient for aggressive or advanced cases. **Table 1** outlines various treatment strategies for distinct types of breast cancer, highlighting the recommended approaches for each.

**Table 1:** Systemic therapeutic strategies for breast cancer.

Therapy	Application	Advantages	Disadvantages	Type of cancer	Possible approach
Resection	Localized tumors Any stage	Allows for an accurate etiological diagnosis.	Risk of surgical complications. Psychological impact.	Luminal	Resection + Hormonal therapy
				HER2+	Resection + chemotherapy + Targeted therapy (Trastuzumab)
				TNBC	Resection + chemotherapy/intensive radiotherapy
Radiotherapy	Post resection Local control	Preserves the breast	Side effects on the skin, heart and lungs.	Luminal	External radiotherapy
				HER2+	External radiotherapy (lymph nodes) + trastuzumab
				TNBC	External radiotherapy (lymph nodes) + resection
Chemotherapy	Neoadjuvant and adjuvant	↓Tumor burden ↓ Metastasis	Side effects due to toxicity	Luminal	Adjuvant, AC-T
				HER2+	Neoadjuvant/adjuvant, AC-T + trastuzumab, TCHP
				TNBC	Adjuvant, AC-T, TC
Targeted Therapy	HER2+ tumors	↑Specific efficacy	Risk of cardiac toxicity Excessive costs	Luminal	CDK4/6 inhibitors mTOR inhibitors PI3K inhibitors
				HER2+	Monoclonal antibodies Tyrosine kinase inhibitors
				TNBC	PARP inhibitors PD-L1-targeted immunotherapy Conjugated antibodies

#### 3.1.2 Radiotherapy

According to the National Cancer Institute, radiotherapy is a cancer treatment that utilizes high-energy beams to destroy or slow down cancer cell growth. There are two primary approaches: external radiotherapy, where radiation is emitted from outside the body, and internal radiotherapy, where a radiation source is administered inside the body (114). Liquid radiotherapy (radiopharmaceutical therapy) is considered a systemic treatment because, when administered intravenously or orally, it can travel throughout the body, allowing it to treat both primary tumors and metastases (115). Radiotherapy works by causing DNA damage in cancer cells, leading to cell death through apoptosis or necrosis (116). Healthy cells have mechanisms to repair the damage, minimizing genetic mutations that could trigger disease in most cases (117).

External radiotherapy is highly effective as an adjuvant treatment for early-stage breast cancer, reducing the risk of local recurrence. Studies have shown that patients with luminal breast cancer treated with radiotherapy experience lower rates of local and regional recurrence and improved overall and local survival. However, it can also harm healthy cells, leading to side effects such as skin reactions, fatigue, and inflammation (118, 119). Rare but severe side effects include lung damage, fibrosis, and secondary cancers (120–123). Recent advances in external radiotherapy have focused on decreasing radiation exposure to surrounding tissues, thereby reducing local and regional recurrence rates (124–127). Various specialized therapies have been developed to meet these goals, improving treatment outcomes for patients, though side effects remain a concern.

#### 3.1.3 Chemotherapy

Chemotherapy is a systemic cancer treatment that aims to inhibit cell proliferation and, consequently, tumor growth (128). The first studies on this treatment date back to the mid-20th century as a result of observations of the effect of mustard gas on soldiers fighting in World War II (129). The discovery of the gas’s myelosuppressive properties ushered in an era of significant advances in chemotherapy development, making it the first line of defense against many types of cancer (130). Different types of chemotherapy can act by damaging the DNA of cancer cells (alkylating agents), mimicking molecules necessary for DNA and destabilizing its synthesis (antimetabolites), inserting flat molecules into DNA to generate an increase in free radicals (anthracyclines), or affecting both the cytoskeleton (microtubule inhibitors) and enzymes (topoisomerase inhibitors) to block mitosis and DNA unwinding (131). Depending on when they are applied, they may be considered neoadjuvant, adjuvant, palliative, or maintenance chemotherapy.

It has been demonstrated that combining chemotherapy with other types of therapies, such as surgery, radiation, hormone therapy, immunotherapy, or targeted therapy, significantly reduces the risk of recurrence compared to chemotherapy alone in various types of cancer (131). Although chemotherapy regimens depend on the type of breast tumor and its stage, there are standard regimens that are commonly used, such as AC-T, which consists of Adriamycin, Cyclophosphamide, and Taxane. This regimen has been shown to be highly effective in multiple studies, reducing recurrence and mortality (132–135). On the other hand, the same qualities that make chemotherapy so effective against cancer also affect healthy cells in breast cancer patients, causing side effects such as fatigue, nausea, vomiting, alopecia, infertility, cognitive impairment, gastrointestinal problems, early menopause, heart problems, and risk of teratogenesis (136–140). The degree of damage caused will depend heavily on the type of chemotherapy used, the type of cancer, and the body’s ability to develop chemoresistance (141).

#### 3.1.4 Endocrine therapy

Hormone receptor-positive (HR+) is the most common breast cancer subtype in both premenopausal and postmenopausal women (60% *vs* 75%, respectively). Endocrine therapy is the primary treatment, with selective estrogen receptor modulators (SERMs) used for premenopausal patients and aromatase inhibitors (AIs) for postmenopausal patients. Studies have shown that AIs are effective in reducing recurrence and contralateral breast cancer risk. Prolonged AI therapy (2–3 years) may be beneficial for patients with high-risk features, despite increased side effects including risk of cardiotoxicity, osteoporosis, fractures, bone pain, hot flashes, myalgia, and arthralgia. Overall, AIs have become a widely used treatment for HR+ breast cancer (142).

Aromatase inhibitors (AIs) are classified depending on their mechanism of action into two types: a) Nonsteroidal AIs (reversible binding), and b) Steroidal AIs (irreversible binding). AIs have evolved through generations: First generation (Aminoglutethimide): nonsteroidal, non-selective, and associated with significant side effects. Second generation (Fadrozole, Formestane): more selective, but efficacy is not superior to tamoxifen. Third generation (Anastrozole, Letrozole, Exemestane): highly selective, strong specificity, and reduced side effects. These advancements have improved the effectiveness and safety of AIs in treating hormone-sensitive conditions, but side effects remain a challenge to adhering to treatment, mainly for pain sensation (143).

### 3.2 Targeted therapy

Targeted therapy, also known as molecular therapy, is a type of cancer treatment that works by inhibiting specific molecules in cancer cells involved in growth, progression, and metastasis (144). The first studies related to this type of treatment were conducted on trastuzumab, an effective treatment for blocking HER2 signaling, which is overexpressed in approximately 20-25% of BCs (145). With the advent of the Cancer Genome Atlas (TCGA) program, advances in targeted therapy were accelerated. Although BC was already classified into clinical subtypes before this program, the TCGA refined the classification based on somatic mutations (PIK3CA, TP53, etc.) and gene expressions (HER2, EGFR, BCR-ABL, RAS), thereby clarifying potential therapeutic targets (146).

Immune checkpoint inhibitors (ICIs) have transformed cancer treatment over the past decade, with approximately 46% of patients in the USA potentially eligible for this therapy. The main goal is to block proteins that cancer cells use to evade the immune system, thereby activating the body’s T-cells to attack cancer cells more effectively. ICIs are monoclonal antibodies targeting CTLA-4–CD28 or PD-1/PD-L1 axes. In breast cancer, ICIs are mainly used in TNBC due to its limited treatment options. In early-stage TNBC, ICIs may be given alongside chemotherapy before surgery (neoadjuvant) and continued after surgery (adjuvant) to reduce the risk of cancer relapse. Despite their therapeutic benefits, ICIs are associated with immune-related adverse events (irAEs) due to excessive immune system activation. The incidence of irAEs varies depending on the agent, cancer type, and patient characteristics. Fatal events are rare (0.3-1.3%), with a median onset time of 14.5 days. Understanding the unique profile of irAEs is crucial for clinicians to manage patients receiving ICIs effectively (147).

Given that BC is strongly associated with exposure to E2, and that its receptors are overexpressed in up to 70% of all cases (148), it is expected that most targeted therapies will aim to inhibit estrogen and HER2 signaling in patients with ER-positive BC (149). To this end, strategies developed from hormones and immune system cells have been widely used. Many targeted therapies for HER2+ BC seek to block it using monoclonal antibodies (150), modify its dimerization to other HER receptors (151) or tyrosine kinase inhibition (152–154), while for the HER2- subtype, the trend is seeks to direct therapy to PI3K inhibitors for tumors with PIK3CA mutations (155, 156), mTOR inhibitors and CDK4/6 (157, 158). These types of approaches, whether used alone or in combination, are more effective in treating breast cancer than other types of therapies (159).

On the other hand, research into triple-negative BC has shown that therapies such as PD-L1-targeted immunotherapy (160–162) and Trop-2-targeted antibody conjugates (163–165) may show a significant improvement in overall survival compared to traditional chemotherapy treatments, especially in the early stages of the disease. In the case of tumors with BRCA1/2 mutations, PARP inhibitors have been shown to significantly reduce the risk of recurrence and increase patient survival (166, 167). Despite the significant benefits of targeted therapies, several challenges persist in current research. These include the development of long-term acquired resistance, high treatment costs, and the need to identify more effective biomarkers (168, 169). Further investigation is necessary to overcome these limitations and optimize treatment outcomes (**Table 1**).

## 4 Intratumoral therapies: a promising approach for BC

Intratumoral therapies in solid tumors have been explored more recently, as they have shown benefits compared to systemic treatments, including the avoidance of toxic effects, reduced drug doses, and activation of the immune response within the tumor (170).

### 4.1 Systemic *vs* intratumoral treatment: a comparative overview

Neoadjuvant and adjuvant therapies are commonly recommended in BC treatment to improve the likelihood of curing many solid tumors. These therapies may include hormonal agents, radio, chemo, or immunotherapy. Neoadjuvant treatment is administered preoperatively and aims to reduce tumor growth and eliminate disseminated cancer cells, while adjuvant therapy is given postoperatively to target micrometastases and increase cure rates. These treatments are typically delivered systemically and can reduce recurrence and mortality, but may also increase the risk of death from non-cancer-related diseases (171–173). Patients with BC who receive these systemic therapies are not exempt from adverse effects associated with such treatments. Common side effects include pain, irritation, redness, fatigue, organ inflammation, and diarrhea, among others. Additionally, it has been observed that systemic administration of immunotherapy often results in suboptimal pharmacokinetic profiles, limiting effective access to tumor cells within the TME (174).

Intratumoral (i.t.) therapy, which dates to the 19th century, has been used to treat different types of cancer (175). This mode of administration allows for the direct delivery of various anticancer therapies (including small molecules, nucleic acids, protein therapies, viral vectors, and cell therapies) into the tumor, achieving high local drug concentrations at the target site, reducing required dosages, and minimizing systemic toxicity by avoiding off-target drug uptake. Moreover, i.t. administration enables drug delivery to poorly perfused tumor regions (176). Intratumoral administration has shown promise as a neoadjuvant strategy, improving outcomes in patients with early-stage solid tumors. *In situ* antitumor therapies are designed to act within the TME, enhancing immunogenicity, promoting tumor-infiltrating lymphocytes (TILs), and reversing cancer-induced immunotolerance, thereby triggering an effective antitumor immune response (177). Recent advances have refined i.t. drug delivery to enhance accuracy and precision using image-guided techniques such as computed tomography, ultrasound, magnetic resonance imaging (MRI), and X-ray fluoroscopy (178, 179). Below, we will describe the most commonly used intratumoral delivery systems, and subsequently, we will discuss the advantages of intratumoral administration that have been successfully employed in BC.

### 4.2 Intratumoral drug delivery systems

Intratumoral drug delivery systems have recently garnered significant attention, with a focus on nanoparticles, hydrogels, microneedles, microbeads, and similar technologies (180). The development of these delivery systems must account for specific physicochemical characteristics of theTME, such as excessive lactic acid production, resulting in an acidic extracellular pH in solid tumors (~5.6), compared to normal tissues (pH 6.5–6.9) (181). **Table 2** describes the intratumoral therapies employed in BC.

**Table 2:** Delivery systems for intratumoral administration in BC: neoadjuvant therapy.

Delivery system	Drug	Cancer *in vivo*	Ref
Hydrogel			
Chitosan	Interleukin-12	4T1cells in female BALB/c nude mice	(182)
Chitosan XCS gel	Interleukin-12	E0771 cells in C57BL/6 mice	(183)
Hydrogel poly (lactic acid-*co*-glycolic acid) (PLGA) and poly (ethylene glycol) (PEG) PLGA–PEG–PLGA	Tamoxifen	MCF-7 cells were injected into nude mice	(184)
Poly (organophosphazene) (PPZ)- PEG-*g*-chitosan hydrogel	2-Methoxyestradiol	Balb/c nude mice MDA-MB-231 breast cancer cells	(185)
Hydrogel	XCS gel/IL-12	Balb/c mice with 4T1 cells	(186)
Nanoparticles and microspheres			
Folate-modified lipid nanoparticles.	System delivery of circular single-stranded DNA expressing IL-12	Balb/c mice with 4T1 cells	(187)
Small interfering RNA nanoparticles	Macrophage migration inhibitory factor (MIF)	Balb/c and C57BL/6J mice with 4T1 cells	(188)
Nanoparticle (NP-SiCD47)	Chemokine CCL25	Balb/c mice with 4T1 cells	(189)
Poly-lactic-acid-encapsulated microspheres (PLAM)	IL12+tumor necrosis factor (TNF-α), +GMCSF	Balb/c mice with 4T1 cells	(190)
Lentivector			
Chitosan + transcription construction	IL12 + GMCSF+IL-21 IL12 + GMCSF+IL-15	Balb/c mice with 4T1 cells	(191)

#### 4.2.1 Hydrogels

These are a three-dimensional biocompatible network of gels capable of loading various antitumor drugs. They offer continuous release and high local concentrations of the drugs within the tumor (180, 181). Hydrogels are biodegradable and can be eliminated after drug release or disintegrate into small fragments for excretion. Common hydrogels include chitosan–beta-glycerophosphate, poly(N-isopropylacrylamide) with natural polymers, poloxamers, and amphiphilic copolymers composed of PEG and polyesters (181).

#### 4.2.2 Nanoparticles and microspheres

Intratumoral drug delivery via nanoparticles enables sustained release and higher drug encapsulation rates (180). Particle size is a critical determinant of tumor penetration and therapeutic efficacy. Nanoparticles ranging from 20 to 40 nm can penetrate deeply into tumor tissues (192). Types of nanoparticles used for BC treatment include metallic, polymeric (both natural and synthetic), hybrid, and carbon-based nanomaterials (193). Microspheres are matrix-based particles that evenly distribute drugs within a polymer matrix. They can encapsulate macromolecules, biomolecules, and nucleic acids. Microspheres range in size from 1 μm to 1000 μm and can provide sustained drug release through hydrolysis, self-diffusion, transport, and erosion (180, 194).

#### 4.2.3 Microneedles

Another potential strategy for drug delivery in BC involves the use of microneedles (MN). These micron-scale needles allow for nearly painless transdermal drug administration and have shown strong potential for a delivery system. They may be combined with nanoparticles or drug-loaded formulations. Upon administration, the microneedles are dissolved into theTME, gradually releasing the drug. Typically arranged in patches, they ensure uniform distribution of the drug across the tumor tissue. Well-designed microneedles enhance the antitumor efficacy of the loaded therapeutics (180, 195). Extensive research in developing new hydrogel-based MN has used hyaluronic acid crosslinked with polyethylene glycol (PEG), providing up to 121 needle projections with only 600 μm in length, proving stability for one year, and the capacity to encapsulate and deliver multiple functional drugs, including proteins, cytokines, small molecules, and even cells. Since the hydrogel appears to shield the entrapped molecules, preventing protein conformation changes, and long-lasting biological activity is a potential tool to deliver directed molecules or even trained cells into the TME (196).

#### 4.2.4 Promising new developments in intratumoral and other delivery systems in BC

**Figure 4:**
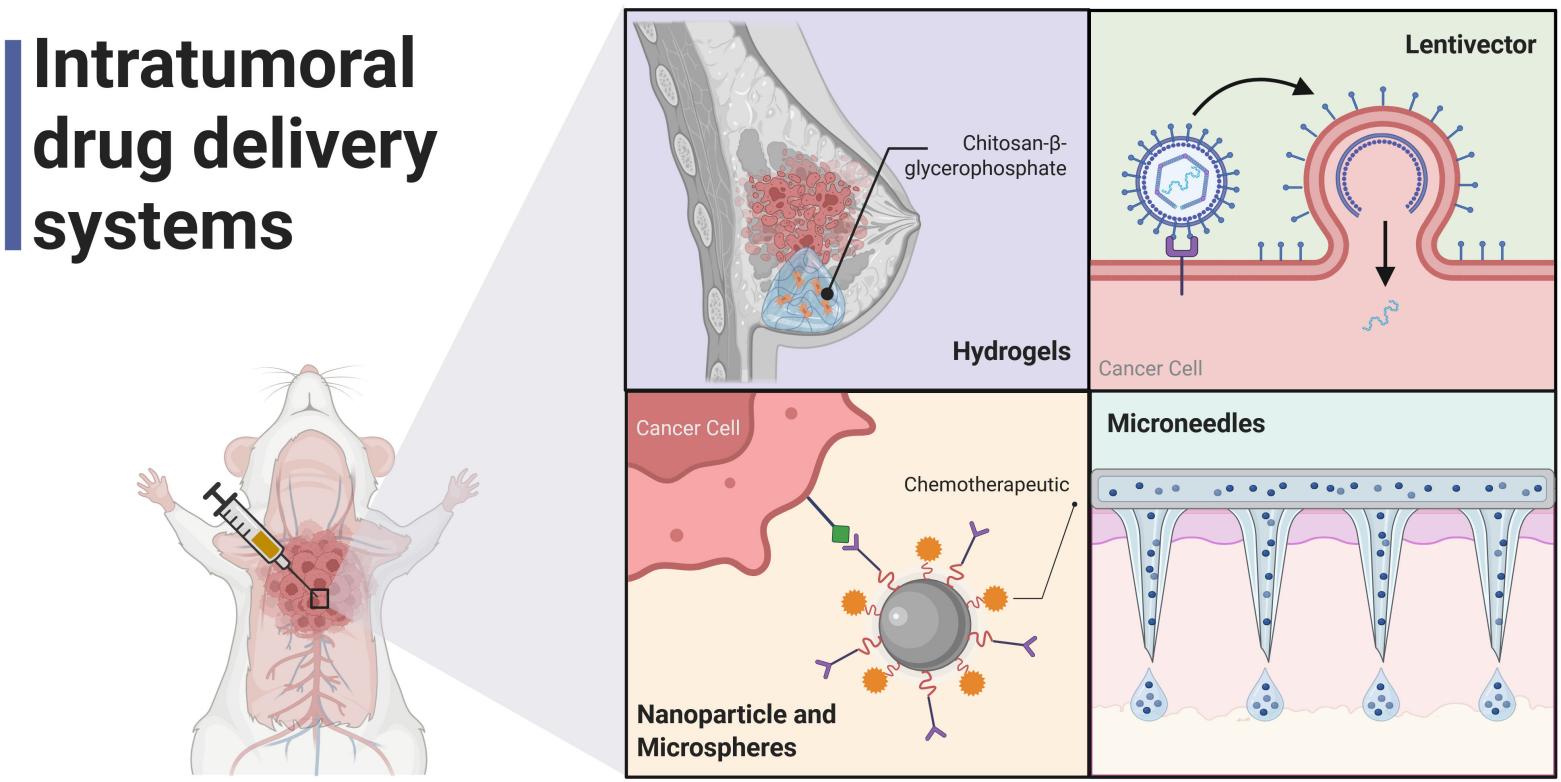
Intratumoral drug delivery systems. image created with Biorender.

Hydrogels and nanoparticles have been among the most used delivery systems for intratumoral administration. However, there is a need to develop additional biomaterials that can enable intratumoral drug release. Currently, several nano delivery systems are being evaluated, including polymeric micelles, liposomes, peptide-drug and antibody-drug conjugates, extracellular vesicles, targeted protein degradation systems, cell- or membrane-coated nanoparticles, and delivery systems based on oncolytic viruses (170). **Figure 4**. A cytokine delivery project of a Hydrogel-based microneedle (MN) patch administered intradermally, composed of hyaluronic acid (HA), has been successfully used to deliver CCL22 and IL-2 for Treg recruitment and expansion, providing long-lasting and uniform delivery, which results in the remodeling of the local milieu. This engineered polymeric microneedle also presents diagnostic potential for sensing interstitial fluid, as its matrix contains disulfide bonds that are prone to dissolve upon retrieval under proper conditions, and isolates cellular biomarkers useful for reporting the disease status (196).

**Figure 5:**
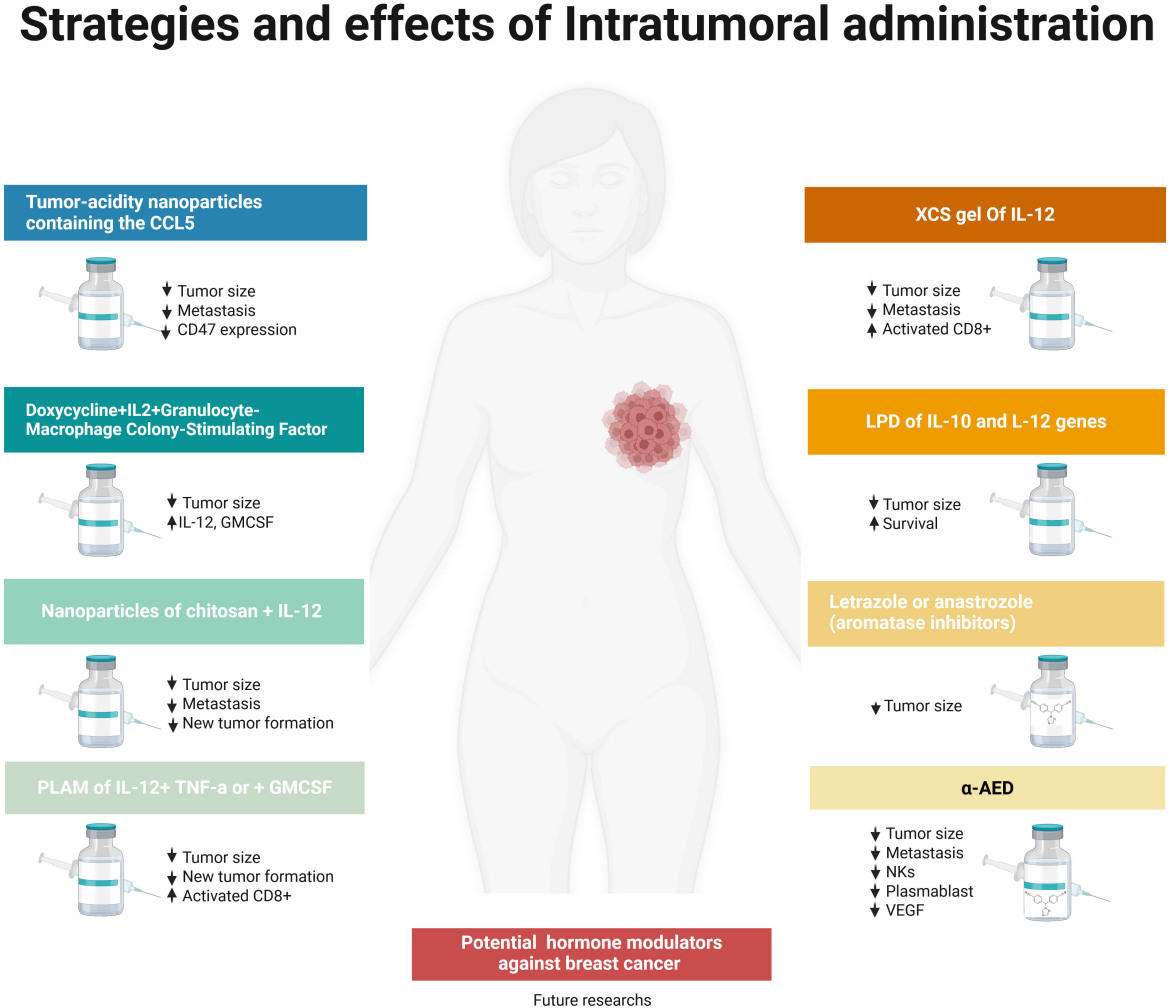
Strategies and effects of intratumoral administration of cytokines and chemokines in BC models. Cytokines and chemokines modulate distinct immune responses within the breast TME, influencing patient prognosis. Various approaches, including nanoparticles, viral vectors, transgenic cells, hydrogels, and biodegradable polymers, have been employed for local cytokine delivery, resulting in tumor regression, activation of cytotoxic T cells, inhibition of metastasis, and induction of immune memory. Combinations including IL-12, IL-2, GM-CSF, and TNF-α have demonstrated significant antitumor efficacy. Intratumoral therapies using aromatase inhibition induce profound tumor regression and outperform systemic treatments like tamoxifen in ER+ BCs. The direct delivery of α-AED reduces tumor burden and metastases while altering the immune microenvironment by increasing NK cells, plasma blasts, and plasma cells, and lowering VEGF levels. These findings underscore the potential of intratumoral therapies not only in hormone-sensitive tumors but also in aggressive, immune-responsive subtypes, including TNBC. These therapies represent promising alternatives to systemic cytokine administration, offering improved specificity and reduced adverse effects. Image created with BioRender.

Other strategies have included the use of free DNA or RNA, or their encapsulation in lipid nanoparticles encoding cytokines, viruses expressing cytokines, and engineered cells designed to produce exogenous cytokines. Liposomes, exosomes, and cytokines conjugated with antibodies have also been explored (197), **Figure 5**.

Intratumoral delivery systems must consider that the vasculature within and around the tumor is complex and that the properties of the TME are altered. The acidic environment in tumors may reduce drug efficacy by impairing the permeation of the drug. For this reason, the features of the TME should be considered when developing these delivery systems (180, 181). Taking these aspects into account in formulation design offers advantages such as improved drug penetration into the tumor, sustained therapeutic response, and reduced side effects. Moreover, not only pH but also reactive oxygen species, light, and heat should be considered. Therefore, an intratumoral delivery system that addresses all of these factors represents a promising strategy to enhance the efficacy of drugs administered directly into the tumor (180).

For this reason, various intratumoral delivery systems have been developed in recent decades. Due to their advantages, they have been used not only to deliver hormones, cytokines, chemotherapy, and immunotherapy agents, but also to incorporate radiosensitizers and photothermal agents, to reduce adverse effects (180). Finally, it is essential to continue research on intratumoral delivery systems for BC to optimize pharmacological effects and minimize toxic side effects for patients. **Table 2**.

## 5 Intratumoral therapies in BC to modify the neuroimmune endocrine network

Most of the drugs administered intratumorally are immune modulators, including immune checkpoint molecules, oncolytic viruses, nucleic acid therapy, innate immune agonists, bacteria, chimeric antigen receptor (CAR)- T cells, NK cells, and dendritic cells, alone or in combination with immune or chemotherapy agents (170, 198). Nevertheless, few studies have addressed the intratumoral administration of components or blockers of the neuroimmune endocrine network. This section aims to expand the knowledge of hormones and soluble factors, including cytokines and chemokines, as well as neurotransmitter blockers that can be administered intratumorally to modify the TME.

### 5.1 Cytokines and chemokines administration in BC

In BC, the metastatic process is the leading cause of high mortality because it is often discovered in late stages of the disease, limiting breast cancer treatment. Of note, metastatic BC cells acquire aggressive characteristics from the TME (199). In the early stage of BC disease, it is now clear that the action of antitumor immunity can prevent tumor progression. Nevertheless, exacerbated proinflammatory cytokine release by the immune cells can induce a quiescent state in various immune cells within the TME and promote tumor metastasis (200). For this reason, the employment of different cytokines and chemokines that reactivate the immune system actions is considered a novel therapeutic option in the intratumoral scheme in BC.

Cytokines and chemokines elicit different actions in different immune cells. Interestingly, it has been reported that specific cytokine and chemokine profiles exist in the tumor breast microenvironment, and these profiles have a strong relationship with patient survival. In this regard, it has been described that high tumor cell differentiation correlated with increased intratumoral concentrations of interferon IFN-α and reduced transforming growth factor (TGF-β1). Additionally, different types of immune responses are linked to distinct cytokine microenvironments and correlate with relevant differences in patient prognosis (201). Regarding the above, systemic administration of cytokines has been successfully employed for BC treatment. Nevertheless, there are still some issues that need to be addressed, including: raising the cytokine specificity for receptor subunits, protecting cytokine stability, favoring the cytokine transport into the circulation, fusing cytokines with tumor- or effector cell-targeting moieties, encapsulation of cytokines in biomaterials or nanoparticles to accumulate into the tumors (197) **Figure 5**.

#### 5.1.1 Intratumoral cytokines in BC

Regarding intratumoral cytokine administration, there are limited studies in BC tumors. More recently, the direct injection of cytokines into the tumors has become a promising therapeutic alternative for cancer treatment. Some modalities that have been implemented for the intratumoral administration of cytokines include cytokine-encoding mRNAs or DNAs alone or contained in lipid nanoparticles (LNPs), virus-encoding cytokines, cytokine-expressing transgenic cells, immune cytokines, recombinant cytokines, and biomaterial-anchored cytokines (absorbed in microneedles) (197, 202, 203). To date, there are few studies on the intratumoral cytokine administration in BC models. Intratumoral cytokine-based treatments for BC will be described below:

Interleukin (IL)-6 is a cytokine with critical pleiotropic actions in BC. This protein has been significantly associated with inflammatory and metastatic processes in breast cancer (204). The IL-6 actions are controversial; for instance, direct application of IL-6 to breast cancer cells inhibits proliferation in estrogen receptor-positive cells, while high circulating IL-6 levels correlated with a poor prognosis in BC patients. The above indicates that local intratumoral IL-6 signaling plays a crucial role in controlling BC cell growth, metastasis, and self-renewal of cancer stem cells (205). However, studies on the intratumoral administration of IL-6 in breast tumors have not been conducted yet.

Interestingly, the administration of cytokine combinations with antineoplastic drugs has been proven. Regarding this point, the intratumoral administration of the combination of Doxycycline+IL2+IL12+Granulocyte-Macrophage Colony-Stimulating Factor (GM-CSF) and chitosan/IL2+ IL12 + GM-CSF, cytokines involved in activation and expansion of T lymphocytes (IL-2), stimulation of other immune cells and antiangiogenic properties (IL-12) and differentiation of immune system cells (GM-CSF), was shown to decrease the tumor size of BC tumors in mice to the extent of tumor regression. The authors postulated that other combinations, such as IL-12 + GM-CSF+IL-21 and IL-12 + GM-CSF+IL-15, had significant effects in the tumor reduction in mouse tumor models. The authors also explained that the lentivector system used for the intratumoral injection is composed of biodegradable materials, such as chitosan, and the transcription construction yielded suitable and promising results for intratumoral treatment (191). Other therapeutic formulations based on particles shaped with chitosan and IL-12 resulted in the inhibition of BC tumors in a mouse model. In addition, the mice that received the chitosan/IL-12 formula were protected from BC metastasis and also completely protected from rechallenge with triple-negative breast cancer cells (TNBC) cells (182).

Other intratumoral combinations with IL-12 probed in BC *in vivo* studies correspond to poly-lactic-acid-encapsulated microspheres (PLAM) containing IL-12 + tumor necrosis factor (TNF-α) + GM-CSF (190). The results demonstrated that a single intratumoral injection of cytokine-loaded PLAM significantly suppressed tumor growth. In particular, the combination of IL-12 and TNF-α facilitated an increase in T CD8 cells infiltrating the tumor and spleen. In addition to tumor rejection after 3 weeks, this treatment generates an immune memory compared to controls. In addition, the IL-12+ TNF-α PLAM treatment resulted in the evasion of tumor formation of a new BC challenge in mice (190). The authors also discussed that slow-release polymer microspheres are excellent vehicles for sustained release of cytokines into the TME (190). The intratumoral administration of IL-12 in BC models has also been proven in a novel injectable hydrogel, called XCS gel (186).

A single intratumoral administration of XCS gel/IL-12 eradicated 86% of BC tumors in mice, as well as inducing significant changes to the tumor-immune microenvironment, such as the increase in activated, proliferating T CD8+ cells. Notably, XCS gel-IL12 was well tolerated with no severe local or systemic side effects (186). A folate-modified lipid nanoparticles system for the delivery of circular single-stranded DNA expressing IL-12 has also been proven for intratumoral treatment in BC models. This delivery system was effective for activating anti-cancer immune responses, diminishing tumor growth, prolonging survival in animal models, and preventing tumor recurrence (187). This novel intratumoral gene therapy is also effective when combined with systemic administration of the anti-VEGFR-2 monoclonal antibody in mice bearing BC cells. This IL-12/VEGFR intratumoral/systemic combination induced significant suppression of tumor growth. In addition, it was also effective against spontaneous lung metastases (206).

The intratumoral administration of IL-10, a major player in controlling the immunosuppressive TME in different tumor models (207), has been tested for the intratumoral delivery of genes encoding an IL-10 protein trap to change the immunosuppressive TME, which could enhance antitumor immunity. It has been reported that the administration of lipid-protamine-DNA nanoparticles loaded with trap genes (IL-10 trap and IL-12 trap) reduced tumor growth and significantly prolonged survival in mice bearing mammary tumors. These immunotherapeutic strategies can be used to prime the immune system, preventing cancer invasion and prolonging patient survival (208). All the above reports indicate that the use of intratumoral therapy targeting cytokine modulation is an important therapeutic strategy that could offer promising results in the treatment of BC.

It is essential to note that cytokines have been utilized in cancer treatment for decades, primarily through systemic administration. Poor therapeutic outcomes and increased side effects have greatly restricted their extensive application (209). For the above, visualizing its intratumoral administration would be convenient since it is effective in reducing tumor growth in BC. A novel mathematical intratumoral modeling approach explains different factors that need to be considered for effective penetration of intratumoral immunotherapy, such as cytokine conjugates. The features for intratumoral administration to be considered include tumor vascular density, permeability, and the tumor hydraulic conductivity, as well as conjugated-cytokines size, binding affinity, and their clearance via the blood vessels and the surrounding tissue, and the interactions with cancer cells (210).

#### 5.1.2 Chemokine administration

Various chemokines have effects that enhance the action of immune cells. In this regard, chemokine (CCL5) acts as a chemoattractant, directing immune cells such as monocytes, lymphocytes, dendritic cells, and eosinophils toward areas of inflammation (211). Regarding this point, an *in vivo* mouse study demonstrated that the intratumoral administration of tumor-acidity nanoparticles containing the CCL5 chemokine, combined with immune therapy targeting the proliferative regulator CD47 protein, enhanced the reduction of CD47 expression. Additionally, the T cell cytotoxic activity and infiltration were examined in a triple-negative BC model. Ultimately, the nanoparticle CCL5/CD47 system led to tumor growth and metastasis inhibition (189).

The CXCL12-CXCR4 axis plays a crucial role in breast cancer metastasis, regulating chemotaxis, migration, and adhesion. CXCR4 expression is higher in malignant breast tumors and controls chemotaxis toward CXCL12, which is highly expressed in organs to which breast cancer cells preferentially metastasize (lung, bone, liver, and lymph nodes). The binding of CXCL12 to CXCR4 and CXCR7 elicits distinct cellular responses, with CXCR4 controlling chemotactic behavior and CXCR7 promoting primary tumor growth and angiogenesis (212). Recent studies have demonstrated that CXCR4 expression is more prevalent in TNBC and correlates with poorer outcomes, highlighting the potential of targeting the CXCL12-CXCR4 axis as a therapeutic strategy. Combining CXCL12 receptor inhibitors with chemotherapy may enhance treatment efficacy and improve patient outcomes by overcoming resistance and promoting survival benefits (213).

Chemokines, such as CCL2, produced by osteoblasts and bone marrow endothelial cells, can promote bone metastasis (214). Additionally, CXCL12-expressing cells in the central nervous system (CNS) act as chemoattractants for metastatic breast cancer cells in the brain. The chemokine CX3CL1, expressed on human bone marrow endothelial cells, interacts with its receptor CX3CR1, which is found on normal and malignant mammary glands. Breast cancer cells with high CX3CR1 expression exhibit a greater propensity for bone metastasis, and studies in CX3CL1-null mice have shown impaired cancer cell dissemination to bone (215, 216). Furthermore, intratumoral administration of inhibitors or monoclonal antibodies raised against these chemokines may offer additional advantages in TNBC, decreasing metastasis, primary tumors, and augmenting overall and disease-free survival in combination with ICIs (1, 217).

Chemokines and their receptors play a crucial role in breast cancer progression and chemoresistance. CCL25, via CCR9, promotes breast cancer cell survival by inhibiting apoptosis through the PI3K/Akt pathway (218). Chemotherapy can also induce chemokine production, creating a feedback loop that enhances chemoresistance. For example, CXCL1 and CXCL2 attract myeloid cells, which produce S100A8/9, promoting cancer cell survival. Alternatively, chemotherapeutic agents induce endothelial TNF-α production, upregulating CXCL1 and CXCL2 in cancer cells, amplifying the CXCL1/2-S100A8/9 loop. Thus, blocking the CXCL1 and CXCL2 receptor, CXCR2, breaks chemoresistance (219). Targeting chemokine receptors, such as CXCR2, CCR5, and CXCR1/2, has shown promise in preclinical models, inhibiting cancer cell invasion, metastasis, and chemoresistance.

Research has shown that CCL5 promotes cancer cell invasiveness through CCR5 signaling. CCR5 antagonists have been found to slow the invasion of basal breast carcinoma cells *in vitro* and reduce pulmonary metastasis in preclinical models, suggesting their potential as adjuvant therapy (220). Additionally, chemotherapy can induce CXCL8 expression, which enhances the activity and self-renewal of chemoresistant cancer stem cells (CSCs). Blocking CXCL8 receptors CXCR1 and CXCR2 may prevent tumor recurrence. Studies have demonstrated that CXCR1 inhibitors not only reduce CSC populations but also decrease overall tumor cell viability (221). The CXCL12-CXCR4 axis plays a significant role in breast cancer metastasis. CXCR4 antagonists, such as CXCL12(P2G) and T140 analogs, have shown promise in inhibiting metastasis in preclinical models (222). These findings highlight the therapeutic potential of targeting chemokine receptors in breast cancer treatment.

Chemokines play a crucial role in enhancing immune cell activity and interactions, making them promising candidates for cancer immunotherapy. CCL19 and CCL21, in particular, facilitate the interaction between dendritic cells and T lymphocytes in lymph nodes, acting as natural adjuvants to boost immune responses. Incorporating these chemokines into DNA vaccines has shown potential in amplifying immunogenicity and inducing Th1-polarized immune responses (223). Oncolytic viruses, another promising cancer treatment, have been enhanced by combining them with CXCR4 antagonists (224). This approach has demonstrated increased efficacy in a triple-negative breast cancer model, inhibiting metastasis and improving tumor-free survival rates.

Nanoparticles have emerged as a promising strategy for targeted cancer therapy. Tumor-targeted nanoparticles can accumulate in tumors, releasing their payload in response to the tumor’s acidic environment. This approach enables efficient cellular uptake and rapid intracellular release. Studies have explored the potential of intratumoral delivery of CCL25 to enhance antitumor immunity. CCL25/CCR9 signaling inhibits the differentiation of CD4+ T cells into regulatory T cells, thereby promoting antitumor responses. Researchers have developed a tumor acidity-responsive nanoparticle delivery system (NP-siCD47/CCL25) that releases CCL25 protein and CD47 small interfering RNA in tumors. In a preclinical model, NP-siCD47/CCL25 significantly increased the infiltration of CCR9+CD8+ T cells, downregulated CD47 expression, and inhibited tumor growth and metastasis through T cell-dependent immunity. Moreover, combining NP-siCD47/CCL25 with PD-1/PD-L1 blockades synergistically enhanced its antitumor effect (189).5.1.3. Chimeric antigen receptor NK and T cells.

Natural killer (NK) cells are linked to better outcomes across solid tumors, including breast cancer, because they can directly lyse malignant cells, secrete IFN-γ/TNF-α to recruit other effectors, and mediate antibody-dependent cellular cytotoxicity (ADCC) via CD16—mechanisms that help contain tumor growth when NKs successfully infiltrate the TME. Therapeutic strategies to potentiate this include (1) killer cell engagers (BiKEs/TriKEs) that bridge CD16 on NKs to tumor antigens to trigger ADCC (2), NK stimulation with cytokine (e.g., IL-2/IL-15/IL-18) to generate NKs with sustained cytotoxicity activity, and (3) approaches that enhance infiltration (e.g., chemokine-receptor engineering or protein-conjugated antibodies) so more NKs accumulate intratumorally where they correlate with improved prognosis (225) In parallel, breast cancer programs are also advancing CAR-T cells against multiple tumor-associated antigens (e.g., HER2, EGFR, c-MET, ROR1, MUC1, GD2), moving from preclinical work into early clinical testing (226).

Chimeric antigen receptor (CAR)–NK cells build on these innate strengths by grafting a tumor-specific recognition domain onto NK cells. Like CAR-T designs, most CAR-NK constructs fuse an scFv to a transmembrane domain and a CD3ζ signaling module, often combined with one or two costimulatory domains (classically 4-1BB or CD28). Importantly, NK-tailored designs that incorporate NK-specific signaling adaptors (e.g., 2B4, DAP10, DAP12) can further boost cytotoxicity and IFN-γ release compared with T-cell–derived architectures, while “armoring” (e.g., membrane-bound IL-15) and memory-like pre-activation improve persistence and function. Together, these platforms expand the toolkit for targeting breast tumors with engineered NK cells. Likewise, breast cancer CAR-T cells use analogous scFv→CD3 costimulation backbones across generations and have been built against the same breast cancer antigens noted above, with trials now exploring feasibility and safety in solid-tumor settings (225, 226).

Systemic NK-cell therapies face practical obstacles in solid tumors: limited *in vivo* persistence after infusion, poor homing through hostile stroma, and suppression within the TME by factors like TGF-β, adenosine, and PGE2. Even when expanded ex vivo, unmodified NKs are typically detectable only briefly post-infusion, and irradiated NK-92–based products persist even less. To increase the number of NKs that actually reach and act within tumors, groups have engineered chemokine receptors (e.g., CXCR1/CXCR4) to follow IL-8/CXCL12 gradients, introduced dominant-negative TGF-β receptors or checkpoint edits (e.g., TIGIT, NKG2A) to resist suppression, and optimized CAR backbones and cytokine support (e.g., mbIL-15, CISH knockout) to enhance durability. Despite these advances, achieving sufficient intratumoral NK density after intravenous dosing remains a central challenge. Similar systemic-delivery limitations apply to CAR-T therapy in breast cancer—imperfect trafficking, an immunosuppressive TME, antigen heterogeneity, and safety concerns—which current strategies address via chemokine-receptor engineering, stromal/ECM targeting, and combination checkpoint blockade (227).

Direct intratumoral (IT) administration can sidestep several of those barriers in breast cancer by depositing effector therapies into the lesion, transforming a “cold” TME into a “hot” one, activating dendritic cells that traffic to tumor-draining lymph nodes, and promoting broader T- and B-cell responses with potential abscopal effects—while also reducing systemic toxicities versus intravenous delivery. Across IT immunotherapy studies in breast cancer, investigators report increased intratumoral cytotoxic lymphocytes (including NK cells), higher CD8+ TILs, and TME remodeling that correlates with clinical responses; importantly, local injection is generally feasible and well-tolerated. However, the use of T and NK cells remains widely unexplored. Then, it is a good opportunity to investigate a potentially more effective treatment, simply by changing the route of administration (228).

### 5.2 Sex hormone-related intratumoral therapies in BC

Intratumoral therapies have become an increasingly relevant area of investigation in hormone-sensitive BCs, particularly ER+ tumors. In these cancers, progression is driven by local and systemic estrogen signaling, which continues even in postmenopausal women due to the extragonadal conversion of adrenal androgens into estrogens via the enzyme aromatase. Importantly, the TME itself can produce local estrogen levels higher than serum levels (229). This makes intratumoral estrogen production a critical therapeutic target. The development of the intratumoral aromatase xenograft model, using aromatase-transfected MCF-7 tumor cells, converts supplemented androstenedione to estrogen, which directly stimulates tumor growth in ovariectomized nude mice. This closely mimics the estrogen-rich microenvironment found in postmenopausal ER+ breast cancer patients (230). Using this model, aromatase inhibitors such as letrozole and anastrozole have shown a dose-dependent suppression of tumor growth.

Letrozole, for instance, at a dose of 10 μg/day, caused nearly 80% tumor regression within 28 days and extended tumor suppression for over 35 weeks, significantly outperforming tamoxifen or even combination regimens (230). Significantly, aromatase inhibitors reduced uterine weight in treated animals, indicating systemic suppression of estrogenic effects, which contrasts with tamoxifen, which maintained or even increased uterine weight due to its partial agonist activity (230). The superiority of intratumoral aromatase inhibition over systemic or combination approaches in estrogen-dependent BC highlights several important insights. First, local estrogen synthesis within the TME is a dominant factor in maintaining tumor growth, especially in the postmenopausal setting. Second, intratumoral administration of aromatase inhibitors ensures a high local concentration of the drug directly at the source of estrogen production, enhancing efficacy while reducing systemic exposure and potential side effects. Finally, the intratumoral model underscores the importance of treatment sequencing: tumors that progress on tamoxifen may still respond to second-line letrozole. At the same time, the reverse is less likely, suggesting that initial therapy choice critically affects long-term outcomes.

While preclinical models have been essential in advancing our understanding of hormone-dependent BC, there are critical limitations that must be acknowledged, particularly when using athymic mice. These mice lack functional T cells due to a congenital thymic defect, which significantly alters the TME (231). As we have discussed extensively, the microenvironment is important in the pathophysiology of tumor development. T cells play a pivotal role in modulating tumor development, immune surveillance, and response to therapy. Their absence in nude mice creates an artificial setting where key immune interactions, especially those involving adaptive immunity, are not represented (231). Another major limitation of current preclinical approaches, particularly those using the intratumoral aromatase model, is their exclusive focus on estrogen receptor-positive tumors, while largely neglecting TNBC. This subtype lacks expression of estrogen receptor, progesterone receptor, and HER2, and is associated with poorer prognosis, higher metastatic potential, and fewer targeted treatment options. Most hormone-based MCF-7 xenograft mouse models are not suitable for studying TNBC, thereby excluding a significant and clinically urgent subset of BC patients from therapeutic innovation.

This is precisely why the study by Ruiz Manzano et al. (2022) gains significant relevance. Unlike most preclinical BC research, which focuses on ER+ models using immunodeficient mice, this work employs a TNBC model in immunocompetent BALB/c mice, allowing for a more accurate representation of the immune-tumor interactions present in patients. The intratumoral administration of 5-androstene-3β,17α-diol (α-AED) significantly reduced tumor size and macroscopic and microscopic lung metastases. Moreover, it modulated the local immune microenvironment, increasing the proportion of NK cells, plasma blast cells, and plasma cells, and reducing VEGF levels—a key driver of tumor angiogenesis and metastasis (232). This study not only demonstrates the potential of α-AED as a therapeutic agent for TNBC but also highlights the effectiveness of intratumoral immunomodulatory treatments in metastatic, aggressive BC subtypes. This area has been severely understudied compared to hormone-responsive tumors.

**Figure 6:**
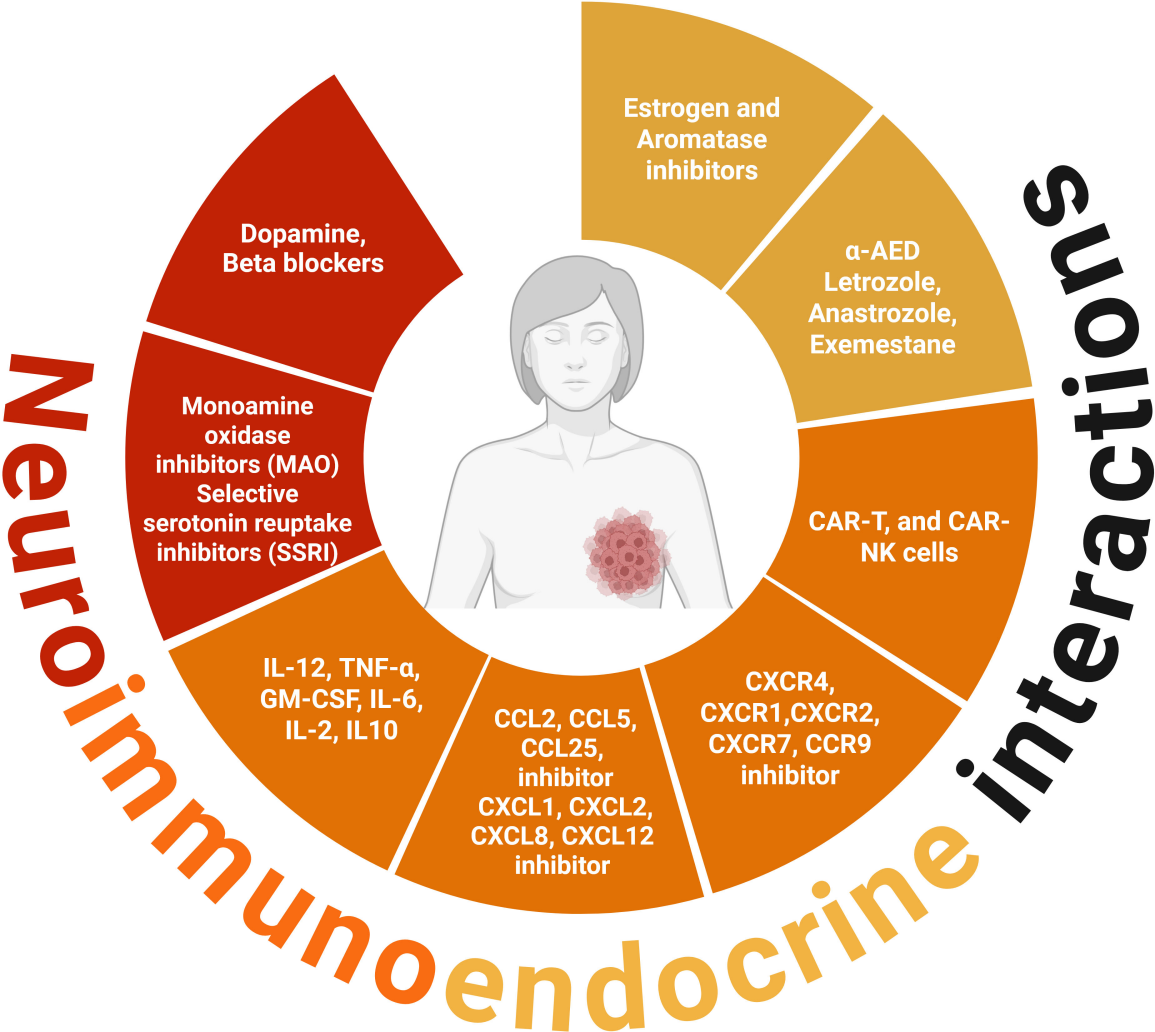
Intratumoral therapeutic strategies targeting the key components of the NIE network signaling in BC models. Image created with BioRender.

This study not only demonstrates the potential of α-AED as a therapeutic agent for TNBC but also exhibits the power of intratumoral immunomodulatory treatments in aggressive BC subtypes, an area that has been severely understudied compared to hormone-responsive tumors. The relevance of these findings is further underscored by emerging evidence on the complex, context-dependent role of androgens and the androgen receptor (AR) in BC biology. AR signaling can exert either tumor-suppressive or oncogenic effects depending on the hormonal receptor context, with anti-proliferative roles predominating in ERα+ settings and more ambiguous, potentially pro-tumorigenic roles in ERα- environments such as TNBC (233). Furthermore, this approach could serve as a conceptual gateway to a broader class of intratumoral treatments leveraging soluble molecules of the neuroimmune-endocrine network, such as neuropeptides, cytokines, and stress-related hormones. These molecules, when delivered directly into the tumor niche, could reprogram local immune responses, modulate angiogenesis, or disrupt tumor-promoting signaling circuits in ways that systemic delivery cannot achieve. It is essential to consider an effective drug delivery system to enhance the long-lasting effects of potential hormone modulators. Altogether, this work lays foundational evidence not only for the repositioning of androgens in breast cancer therapy but also for the emergence of a new paradigm in which tumor-localized modulation of neuroimmune-endocrine pathways becomes a strategic frontier in cancer treatment. **Figure 6**.

### 5.3 Neurotransmitter-related intratumoral therapies in breast cancer

As previously mentioned, breast tumors are highly metabolic and energy-consuming, breaking the homeostasis of the neuroimmune endocrine network. The systemic administration of ß-blockers or antidepressants for other means has been shown to affect tumor progression. Here, we highlight some potential strategies to avoid relapse of BC and metastasis. Cancer patients treated with ß-blockers show better outcomes for breast, prostate, and colorectal cancer; also, cardiovascular patients on ß-blockers resulted in a 49% decrease in cancer risk. A murine model demonstrated that ß-blockers can reverse immunosuppression, enhance the outcome of checkpoint inhibitor chemotherapy, and modulate the activation of MDSCs, as well as their expression of arginase-1 and PD-L1. Neoadjuvant therapy with ß-blockers abolishes the postoperative immune suppression and reduces the risk of tumor metastasis by increasing the infiltration and NK cell toxicity (234).

Antidepressant drugs, particularly monoamine oxidase inhibitors (MAOIs) and selective serotonin reuptake inhibitors (SSRIs), have been found to exhibit anti-tumor properties by modulating the immune system. MAOIs, which increase the availability of serotonin by inhibiting monoamine oxidase, have been shown to enhance anti-tumor T cell activity and depolarize immunosuppressive tumor-associated macrophages (TAMs). SSRIs, such as fluoxetine, have also been found to inhibit tumor growth through their immune-modulatory actions, including the modulation of monoaminergic systems. Specifically, fluoxetine inhibits tumor growth and suppresses cancer progression by restoring NK cell activity, reducing macrophage polarization, promoting T lymphocyte infiltration, and upregulating IFN-γ and granule enzyme B (GzmB) levels, also suppressing the migration and proliferation of tumor cells. However, the use of MAOIs in anti-tumor therapies is limited due to their potential to induce aggressive behavioral side effects. Recent studies have investigated the development of nanoformulations to optimize the administration of MAOIs and mitigate their side effects. The intratumoral administration with proper delivery systems presents an opportunity to increase the anti-tumoral effect (235, 236).

Dopamine (DA) has been shown to exert a protective role in cancer patients, with epidemiological studies indicating that decreased dopamine levels are associated with higher cancer incidence rates. Interestingly, dopamine receptor (DRD1) agonists have been found to elicit a significant anti-tumor effect in preclinical models, despite the receptor’s controversial role in promoting tumor growth and inhibiting immunosuppression. Furthermore, D1-like receptor agonists have been shown to potently inhibit the suppressive function of MDSCs, suggesting that dopaminergic signaling modulates tumor growth by enhancing anti-tumor immunity (237). However, patients treated with DRD2 antagonists have an increased incidence of BC, suggesting different roles of DRD2 (238).

Tramadol has been found to interact with serotonin receptors, and research suggests that it may have anti-tumor effects in breast cancer. Studies have shown that tramadol use after breast cancer surgery is associated with reduced risk of recurrence and mortality. Additionally, antagonists of serotonin biosynthesis and activity have been found to decrease breast cancer stem cell viability, and high serotonin production in tumors may be a predictor of poor prognosis (239). However, the impact of medications that raise serotonin levels, such as selective serotonin reuptake inhibitors (SSRIs), on breast cancer risk and outcomes is unclear. Research suggests that the SSRI fluoxetine may increase breast cancer brain metastases, potentially due to inflammatory changes in the brain (240). A recent study of 7,000 patients in Israel found that SSRI use before or after breast cancer diagnosis was associated with significantly higher mortality rates (241). However, other studies have yielded conflicting results.

Researchers have found that antagonists of serotonin biosynthesis and activity, including certain antidepressants, can target breast tumor-initiating cells (BTICs) across various breast cancer subtypes. Specifically, inhibitors of tryptophan hydroxylase 1 (TPH1), serotonin reuptake transporter (SERT), and certain serotonin receptors have been shown to compromise BTIC activity. Human breast tumor cells express TPH1, serotonin, and SERT, regardless of their molecular or clinical subtype. Treatment with selective serotonin reuptake inhibitors (SSRIs), such as sertraline (Zoloft) and vilazodone (Viibryd), has been found to reduce BTIC frequency and shrink breast tumors in preclinical models, particularly when combined with chemotherapy (242).

Monoamine oxidase A (MAOA) is an enzyme that regulates the availability of neurotransmitters like serotonin and dopamine. MAOA inhibitors (MAOIs), already approved for treating depression and Parkinson’s disease, have shown promise in cancer treatment. Research has demonstrated that MAOA promotes cancer progression and immune suppression, inducing the epithelial-to-mesenchymal transition (EMT) and stabilizing the transcription factor HIF1α (243), while MAOA inhibition can reduce tumor growth, increase macrophage infiltration, and enhance antitumor immunity (244). Wang et al. demonstrated that monoamine oxidase A (MAOA) promotes immunosuppressive polarization of tumor-associated macrophages, hindering antitumor immunity. Inhibiting MAOA with MAOIs suppressed tumor progression in preclinical models and showed synergistic effects when combined with anti-PD-1 therapy. Notably, high MAOA expression correlated with poorer survival in various cancers, including breast cancer. These findings suggest that repurposing MAOIs may enhance antitumor immunity and suppress metastasis.

## 6 Discussion

Breast cancer is a heterogeneous disease that affects millions of women worldwide. An in-depth examination of the cells and soluble molecules involved in each type of breast cancer, as well as an understanding of the interactions within the NIE network, will enable earlier and more accurate diagnostics. The integration of cutting-edge technologies, such as intratumoral administration via hydrogels, nanoparticles, and microspheres, can enhance molecular stability and minimize severe side effects associated with fatalities. Furthermore, the utilization of microneedles enables not only the targeted delivery of drugs and cells but also the detection of specific cells and molecules within the TME. This approach provides valuable insights for the development of more targeted and effective therapeutic strategies. **Figure 6** shows some key components for therapy.

Immune checkpoint blockade therapy has emerged as a crucial tool in treating various cancers, including triple-negative breast cancer. Researchers are actively exploring ways to enhance the efficacy of immunotherapies by modifying the TME to prevent resistance. The success of the therapies relies on the recognition of the communication of breast cancer cells with their surroundings through hormones, cytokines, and recently also through neurotransmitters, which are crucial factors that can impact immune cell function, metastasis, and resistance to new therapies; the impact of this network is regardless of the subtypes of breast cancer.

This review highlights the most significant types and effects of intratumoral treatments, noting that most of these treatments are not yet approved for clinical practice and that complementary studies are necessary to implement them in patients. Extensive research into the TME has paved the way for developing effective personalized medicine approaches, enhancing patients’ quality of life. These findings imply that combining hormone blockers, immune checkpoint inhibitors (ICIs), cytokines, chemokines, or their inhibitors, and antidepressants may offer a promising strategy to overcome breast cancer cell resistance and ultimately reduce mortality rates.
